# Magnetic Particle Imaging in Human Subjects

**DOI:** 10.21203/rs.3.rs-8825005/v1

**Published:** 2026-03-17

**Authors:** Erica E. Mason, Olivia C. Sehl, Marcela G. Weyhmiller, Bryanna Davison, Eli Mattingly, Justin J. Konkle, Toby Sanders, A. Rahman Mohtasebzadeh, Elliott Barcikowski, Kyle Fields, Chris A. Raanes, Nitara Fernando, Carlos M. Rinaldi-Ramos, Osama Mawlawi, Paula J. Foster, Andreas M. Loening, Eric M. Padua, Stephen Y. Lai, Max Wintermark, Patrick W. Goodwill

**Affiliations:** 1 Magnetic Insight Inc., Alameda, CA; 2 Department of Medical Biophysics, Western University, Robarts Research Institute, London, ON; 3 Department of Chemical Engineering and J. Crayton Pruitt Family Department of Biomedical Engineering, University of Florida, Gainesville, FL; 4 Department of Imaging Physics, University of Texas MD Anderson Cancer Center, Houston, TX; 5 Department of Radiology, Stanford University, Stanford, CA; 6 Department of Head and Neck Surgery, University of Texas MD Anderson Cancer Center, Houston, TX; 7 Department of Neuroradiology, University of Texas MD Anderson Cancer Center, Houston, TX

## Abstract

Magnetic Particle Imaging (MPI) is a tracer-based medical imaging modality that detects magnetic nanoparticles with no background tissue signal. MPI acquires quantitative, high-sensitivity tomographic images of shelf-stable magnetic tracers that safely produce signals *in vivo* for weeks or even months. These features can fill capability gaps in medical imaging for applications benefiting from tracer specificity with an extended imaging window. Despite two decades of preclinical validation and multiple published human-scale imagers, MPI has not previously been demonstrated in human subjects. Here we report MPI imaging in two subjects following subcutaneous administration of magnetic tracer in the scalp and foot. Our results showed quantitative and longitudinal visualization of lymphatic drainage for up to six months, with supporting validation in a mouse model. Imaging in human subjects required the development and verification of a novel clinical imager, including magnetostimulation threshold testing of all magnetic fields used in imaging sequences. To understand MPI in the context of existing medical imaging technologies, we benchmarked MPI imaging performance against SPECT using lymphatic system phantom models. These findings demonstrate that MPI can translate from animals to human subjects, and establish MPI as a new tool for longitudinal tracer imaging in medicine. The addition of MPI to the clinical imaging toolbox could enable new approaches and capabilities for diagnosis, real-time interventions, and treatment monitoring across a broad range of clinical applications.

## Introduction

Magnetic Particle Imaging (MPI) is a tracer imaging modality that detects magnetic nanoparticles with no signal from biological tissue. MPI fills a capability gap of existing clinical imaging by sensitively, specifically, and quantitatively imaging a magnetic tracer for weeks to months^1,2^. This unique combination of capabilities contrasts with radiotracer-based imaging, where hour-long decay half-lives can constrain applications and workflows. For example, MPI has demonstrated preclinical visualization of sentinel lymph nodes for days to weeks^3^, and may provide an alternative to the constrained imaging windows used in lymphoscintigraphy requiring careful coordination of tracer injection, imaging, and surgery^4–6^. MPI excels at long-term tracking of adoptive cell therapies including T cells^7,8^, natural killer cells^9^, dendritic cells^10^, and stem cells^11,12^. The technology has also been demonstrated for immune and inflammation imaging^13,14^, targeted imaging of checkpoint inhibitor receptor density^15^, treatment planning for magnetic hyperthermia^16,17^, functional brain imaging in rats^18^ and non-human primates^19,20^, interventional imaging in human leg cadaver specimens^21^, and even directly detecting endogenous hemosiderin to identify unstable plaques^22^.

**Extended Data Figure 1:**
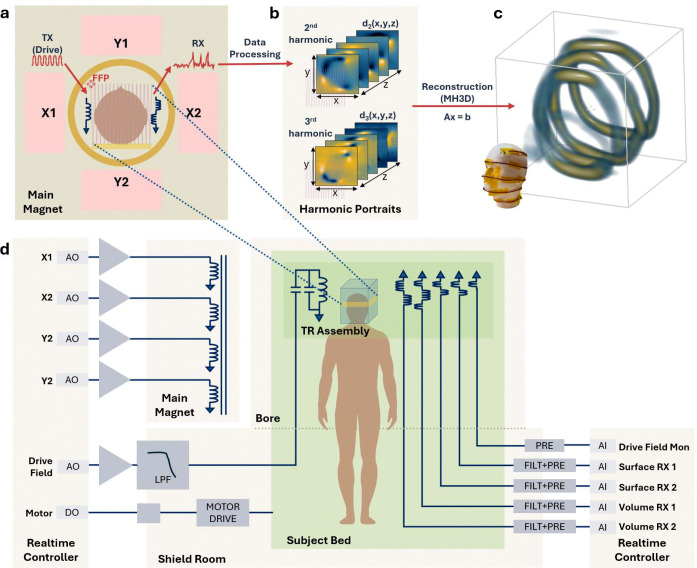
MPI image formation and system diagram. **(a)** To sample MPI signal over a volume of interest, the selection field (here a field free point, FFP) is generated and scanned across the sample by the main magnet (consisting of coils X1, X2, Y1, Y2), while the time-varying drive field (TX) induces the nonlinear magnetization of the tracer particles, whose signal is picked up inductively as voltage in receiver coils (RX). **(b)** The data is processed into an intermediate data format known as ‘harmonic portraits’ (dk(x,y,z), k=2,3...) for each received signal, at each z position of the patient bed. **(c)** The MH3D reconstruction (see Methods) implements a multi-frame deconvolution of the harmonic portraits to form a 3D image of the tracer distribution. **(d)** A simplified system diagram illustrating control and signal flow starting with the real-time controller of the main magnet, drive, and bed motor. The magnetic fields produced by the main magnet and drive coil interact with the magnetic tracer in the field of view, with active control of the drive field amplitude and phase (Drive Field Mon). The magnetic tracer signal is then received, filtered, amplified, and digitized by the same real-time controller.

First published by Gleich and Weizenecker^23^ and in patent literature^24^, MPI measures the nonlinear magnetization response of magnetic tracers such as superparamagnetic iron oxide nanoparticles (SPIOs). A selection field gradient magnetically saturates the tracer throughout the imaging volume except in a small field-free region where time-varying drive fields cause unsaturated magnetic tracer to traverse a nonlinear magnetization curve and induce a voltage signal in receiver coils^23,25^. Magnetic saturation outside the field-free region ensures that signals originate only from the field-free region, which is scanned across the sample to generate images (Extended Data Figure 1a). Signal intensity is linearly proportional to magnetic tracer concentration, providing both quantitative accuracy and tracer specificity with no background signal^26^. These magnetic tracers maintain a signal over weeks to months, enabling sustained longitudinal studies that eliminate the temporal constraints of radiotracer-based imaging^27^.

MPI in human subjects has not been previously published, despite two decades of preclinical validation, human-scale imager development, and basic safety testing^28^. Here we report imaging of the head-and-neck and foot in two human subjects on a novel clinical MPI scanner following subcutaneous administration of magnetic tracer, and demonstrate quantitative, longitudinal lymphatic imaging showing tracer localization in lymph node regions. Human subject imaging was enabled by key technical innovations in the scanner design and verification of the system including subject-specific magnetostimulation threshold testing. The human imaging results are additionally supported by a phantom study to benchmark MPI performance against clinically-available modalities, and a mouse study to validate the translation of MPI from preclinical to clinical. In total, the results reported here validate MPI as safe and clinically translatable, with lymphatic imaging as the first demonstrated application.

## Results

**Extended Data Figure 2:**
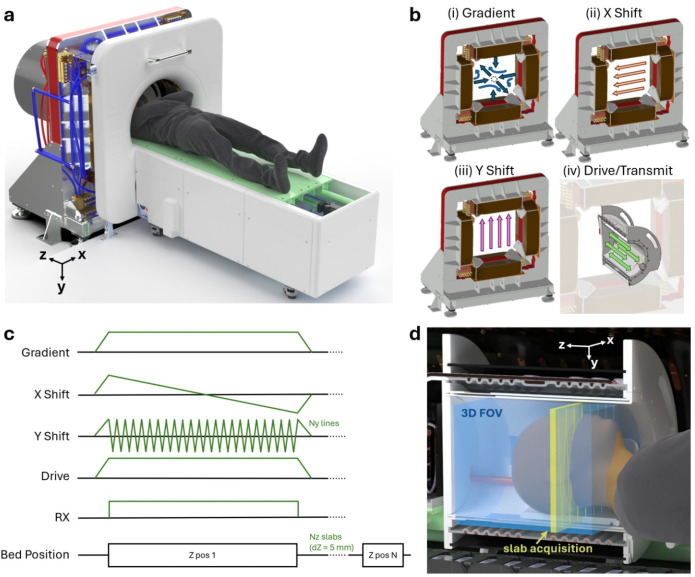
Overview of human MPI hardware and imaging sequence. **(a)** A CAD rendering of the MPI imager shown without shield room walls illustrates the main magnet structure, bore tube, patient bed, and a representative position of the subject during imaging, with their head inside the imaging bore. **(b)** Each of the applied magnetic fields is illustrated: the gradient, Y shift, X shift, all produced by the main magnet, and drive (transmit) field. **(c)** A representative pulse sequence is used to acquire data from a slab, which is repeated at Nz bed positions to image the full FOV. **(d)** A CAD rendering cross-section shows the human head positioned within the transmit/receive assembly, with the slab acquisition from (c) represented in yellow, and the maximum FOV (24 × 24 × 30 cm) in blue. This example pulse sequence (c,d) scans the field free point in the xy plane using a triangular shift waveform with fast shift y and slow shift x. The drive field rapidly moves the field free point in z, defining the slab thickness. The subject bed is stepped along z to acquire multiple overlapping slabs, enabling the full axial FOV coverage.

**Extended Data Figure 3:**
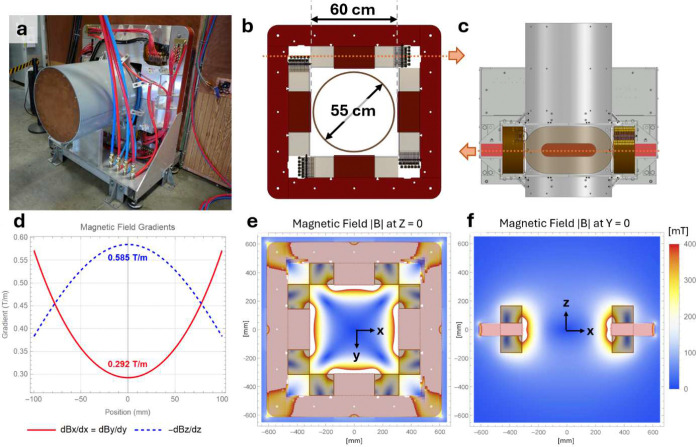
Main magnet assembly detail. **(a)** Close-up of main magnet assembly with the water-cooling (blue and red hoses) visible. **(b)** Axial cross-section through the magnet isocenter and **(c)** coronal cross-section through the top electromagnet show the coil and yoke configuration, highlighting the 60 cm main magnet bore and 55 cm free bore. Orange dotted lines in (b) and (c) show corresponding cross-section locations. **(d)** Simulated magnetic field gradients at isocenter. **(e)** A simulated magnetic field map shows the fields present in the iron yoke and free bore in axial (z = 0) and **(f)** coronal (y = 0) views.

**Extended Data Figure 4:**
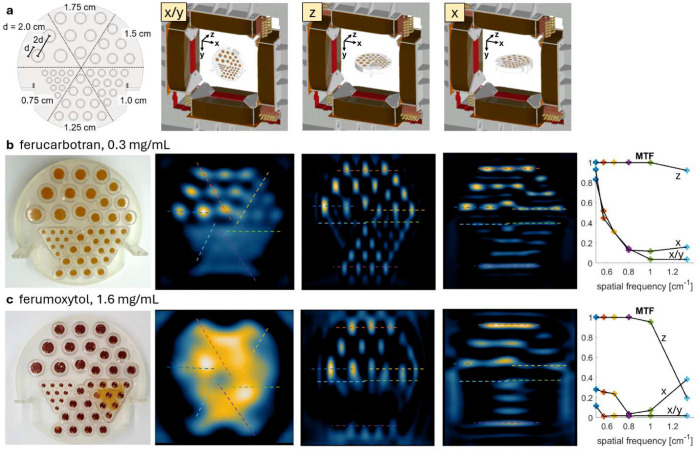
Measured modulation transfer function (MTF) resolution using a Derenzo phantom filled with ferucarbotran and ferumoxytol at a gradient strength of dBx × dBy × dBz = 0.3 × 0.3 × 0.6 T/m. Data points used in the MTF calculation are illustrated as line ROIs overlaid on the 2D slices. **(a)** The phantom was 3D printed and had well sizes ranging from 0.75 cm to 2.00 cm, with 0.70 cm depth. Tomographic volumes were acquired for multiple phantom orientations to capture the anisotropic imager resolution. **(b)** Ferucarbotran resolution was measured to be 1–1.25 cm in x/y (20% MTF) and 0.75 cm in z (90% MTF). **(c)** Ferumoxytol resolution was measured to be >2 cm in x/y and 1.25 cm in z (20% MTF). The leakage seen in the 1.0 cm wells does not impact the results since it is below the resolvable resolution of the tracer.

**Extended Data Figure 5:**
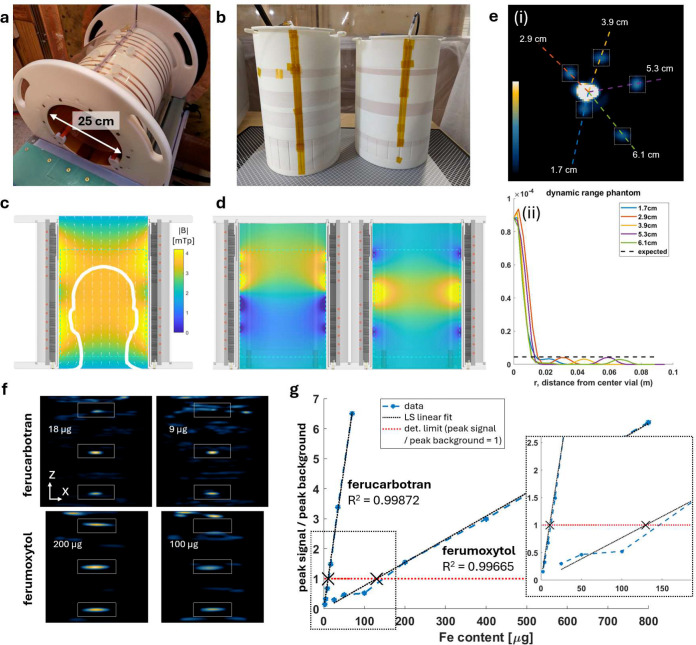
Performance characterization of a head-and-neck volume transmit/receive (T/R) coil assembly. **(a)** Photo of the as-built T/R assembly showing the solenoidal transmit windings and the 25 cm imaging free bore. **(b)** Photo of the two volume receive coils that are mounted concentrically inside the transmit coil in (a). **(c)** Sagittal cross-section of the T/R assembly showing the drive magnetic field magnitude (|B|), and **(d)** the two volume receive coils’ sensitivity maps (Bz component). **(e)** (i,ii) MPI of a shine-through phantom comprised of a central sample containing 2.8 mg Fe surrounded by 5 samples containing 140 μg Fe, demonstrating >20× dynamic range at distances of 1.7–6.1 cm in X/Y plane. The tracer quantities were selected to match phantom studies performed using handheld magnetic probes (see Methods). **(f)** Sensitivity measured using a dilution series estimates **(g)** detection limits of ~10 μg for ferucarbotran and ~130 μg for ferumoxytol. The MPI signal was linear with tracer quantity (ferucarbotran R^2^ = 0.999, ferumoxytol R^2^ = 0.997).

**Extended Data Figure 6:**
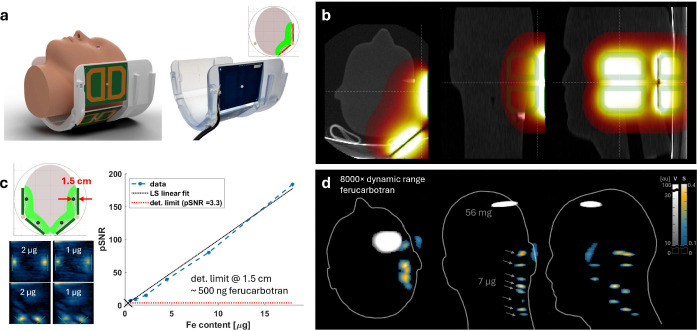
Design, construction, and characterization of an application-specific sur-face coil array designed to improve sensitivity and shine-through performance. The surface coil array can be configured in left, right, or bilateral configurations. **(a)** Render and photograph of the array in its left-side configuration. **(b)** Contour maps show the left-side configuration field maps in axial, coronal, and sagittal views overlaid on an X-ray CT. **(c)** A dilution series sensitivity assessment of the surface coils is performed using the bilateral configuration, showing a detection limit of ~500 ng ferucarbotran at a distance of ~1.5 cm from the coil surface. **(d)** To test shine-through performance, we imaged a lymph node phantom (see Figure 4) filled with ferucarbotran at an 8000:1 ratio in tracer quantity between mock injection site (56 mg Fe) and mock lymph nodes (11×, 7 μg Fe each) and found that 10 of 11 mock nodes were resolvable. MPI axial, coronal and sagittal MIPs are displayed using two colormaps with volume coils displayed in grayscale and surface coils in color.

**Extended Data Figure 7:**
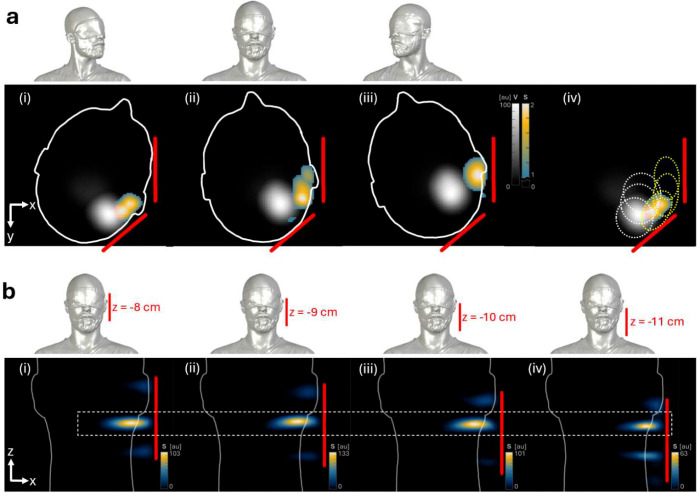
Confirmation of signal in lymph node regions. **(a)** In a first test to confirm that the signals at the lymph node regions maintain a consistent anatomical position within a subject’s head anatomy, a series of images (i, ii, iii) were acquired while the subject rotated their head through three positions. MPI axial MIPs from the crown through the laryngeal prominence are displayed. MPI signal detected from volume coils (V) shows the injection site and is displayed in grayscale. MPI signal from surface coils (S) show the lymph node regions displayed in color. The red lines indicate the position of the surface coils. (iv) The localization of the injection site and lymphatic signals at all three subject positions are summarized with dotted circles, showing the rotation of the MPI signals following the subject anatomy. **(b)** In a second test, a series of images were acquired while the surface coils were adjusted to four positions (i, ii, iii, iv) along the superior-inferior axis of the subject head while the subject position was maintained. MPI coronal MIPs are displayed. In each image, MPI signal was detected in the same cervical lymph node region despite repositioning of the surface coil, showing that the signal maintains a consistent anatomical position regardless of the surface coil position. Slight variation in the images is observed due to interaction between the surface coil’s inhomogeneous receive sensitivity and reconstruction algorithm.

**Figure 1:**
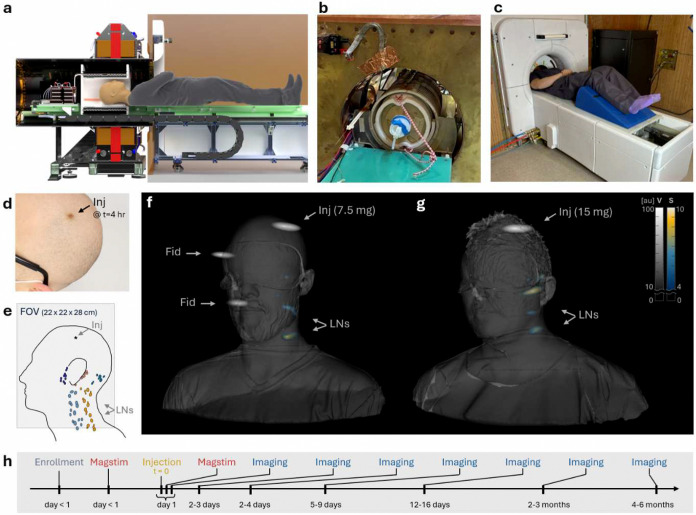
First-in-human magnetic particle imaging demonstration. **(a)** A head-and-neck MPI system was constructed and **(b,c)** verified for basic safety and against FDA criteria for significant risk investigations in MRI. **(b)** Specific absorption rate (SAR) testing was performed on phantoms. **(c)** Magnetostimulation threshold testing was conducted on the head (n=4) and foot (n=6, not shown) for all magnetic fields used in MPI sequences. **(d)** In a subset of subjects, tracer was injected subcutaneously in the scalp (n=2) and in the foot (n=2, not shown). **(e)** For the scalp injection, MPI images are acquired with FOVs that extend from beyond the crown to the laryngeal prominence, allowing imaging of the expected drainage paths in the periauricular and cervical regions^4,31–33^ (illustration adapted from Gregoire *et al.* 2013)^31^. **(f,g)** Tomographic MPI images were generated by compositing data from multiple receiver coils, with volume coil images (V) shown in grayscale and application-specific surface coil images (S) shown in color (see also **Supplemental Video SV1, Supplemental Data SD1,SD2**). MPI volume renderings were co-registered with de-identified surface scans acquired using a hand-held 3D camera. **(f)** Image of subject with 7.5 mg ferumoxytol injection at t = 12.4 days, with two fiducials (Fid) containing 0.5 mg ferumoxytol. **(g)** Image of subject with 15 mg ferumoxytol injection at t = 28.5 hours, no fiducials used. **(h)** Representative time-line illustrating injection (t = 0), magnetostimulation before and after injection, and approximate imaging timepoints up to 6 months post-injection.

To translate MPI to human scale, we designed, built, characterized, and verified the basic safety of a human MPI imaging system, and longitudinally imaged human subjects following magnetic tracer injection (Figure 1, Extended Data Figures 1-7). Basic safety (defined in Methods) testing included SAR testing in phantoms (Figure 1b) and magnetostimulation threshold testing in 6 healthy subjects (4 heads–Figure 1c; 6 feet–not shown). We subcutaneously injected ferumoxytol, an FDA-approved^29,30^ SPIO, into 2 healthy subjects (2 heads–Figure 1d,e; 2 feet–not shown). Tomographic MPI imaging was performed and co-registered with optical surface scans for both subjects (Figure 1f,g; **Supplemental Video SV1; Supplemental Data SD1,SD2**), at time points up to six months after injection (Figure 1h). The head-and-neck images showed magnetic tracer at injection sites and at lymph node regions within the field of view. Each subject had a unique lymphatic drainage pattern to periauricular and cervical lymph node regions, which agrees with known lymphatic anatomical pathways from the parietal scalp^4,31–33^. Both subjects showed signal in the cervical lymph node region that was confirmed by repeated imaging with the subject rotating their head and by moving the surface coils superior-inferior (Extended Data Figure 7). The system achieved sufficient dynamic range and shine-through performance to visualize injection sites and lymph node regions simultaneously. The imaging procedures, with each scan lasting up to 20 minutes, were well-tolerated with no adverse events related to the use of the clinical imager. Tracer administration resulted in expected mild skin discoloration^34^ at all injection sites (Figure 1d, Extended Data Table 2a, Supplementary Figure S1) and resolved within weeks. One subject (S04, 1 mL injection volume) experienced mild pain during injection in the parietal scalp, which resolved immediately (Extended Data Table 2a).

**Figure 2:**
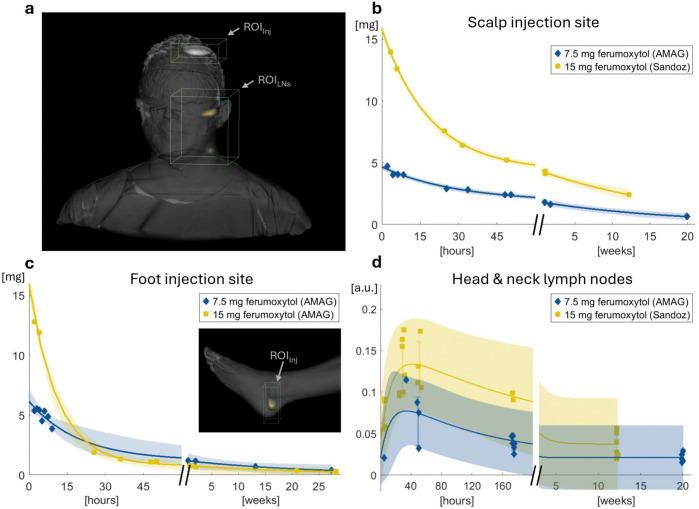
*In vivo* quantitative pharmacokinetics of iron oxide tracer in two healthy subjects. Data are displayed for 7.5 mg dose (blue diamonds) and 15 mg dose (yellow squares) and fit to a bi-exponential (solid lines) with 95% prediction intervals (shaded region). Time constants are reported in Extended Data Table 2. **(a)** Representative MPI image showing 3D regions of interest (ROIs) for quantification of injection site (Inj) and draining lymph node regions (LNs). **(b)** MPI signal at the scalp injection site over time. Data was calibrated with phantoms containing known iron concentrations. **(c)** Calibrated MPI signal at the foot injection site, with inset showing 3D ROI on a representative MPI scan of the foot. **(d)** MPI signal of accumulation and persistence in draining lymph node regions from the head injection. Because of varied scanning sequences and changing surface coil positioning during these exploratory first-in-human scans, lymphatic signal exhibits image-to-image variation and is presented in arbitrary units (a.u.) without calibration. Draining lymph node regions from the foot were unable to be positioned within the scanner’s field of view.

To assess MPI’s unique longitudinal and quantitative capabilities, we quantified the pharmacokinetics of the tracer in the head and foot within regions of interest for up to six months (Figure 2, Extended Data Table 2). Following subcutaneous injection in the parietal scalp, MPI signal was detected at the injection site, which cleared over time (Figure 2b), and in periauricular and cervical lymph node regions, which showed an increase in signal for 48 hours and then a gradual decay over several weeks (Figure 2d). After tracer injections in the foot posterior to the lateral malleolus, signal was detected at the injection site and decayed over time (Figure 2c), but no signal from the lymph nodes is reported as the draining popliteal and inguinal lymph node regions could not be positioned within the scanner’s field of view. The MPI signal decay at both injection sites and head and neck lymph node regions fits well to bi-exponential kinetics characterized by fast and slow phases (best-fit parameter estimates reported in Extended Data Table 2b). At each anatomical location, the higher-dose injection site signal exhibited a faster initial clearance rate than the lower dose, potentially due to the larger injection volume. At each dose, the foot injection site signal exhibited a faster initial clearance rate than the scalp.

**Figure 3:**
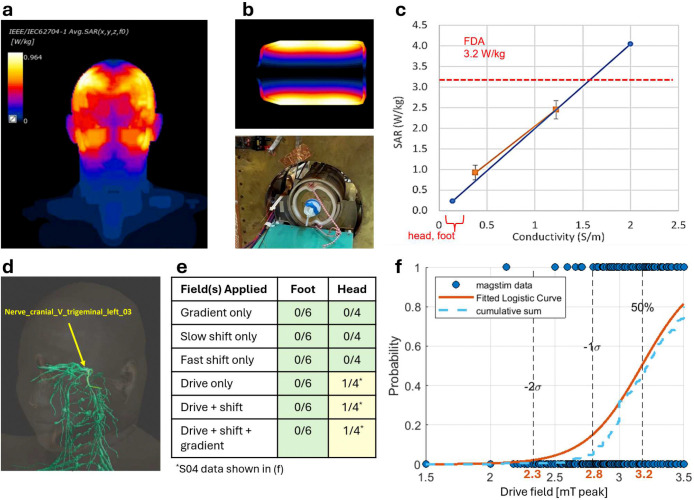
Testing clinical MPI system for basic safety (defined in Methods) and FDA risk guidance for MRI. **(a)** We simulated SAR in the “Duke” virtual body model with a 7 mT peak drive field, which showed an average regional SAR in the head of 0.232 W/kg and a peak SAR of <1 W/kg, both of which are well below the FDA significant risk guidelines for MRI. **(b)** We also measured and simulated SAR in a cylindrical test phantom and **(c)** found good correspondence between the simulation and measurement. **(d)** Magnetostimulation simulation in the “McIntyre-Richardson-Grill” neuronal model predicts peripheral nerve stimulation (PNS) at the cranial V trigeminal nerve at a drive field amplitude of 2.8 mT peak. **(e)** The magnetostimulation test protocol was performed on healthy subjects (n=6 feet, n=4 heads). To determine subject-specific magnetostimulation thresholds and set scanning parameters for imaging, our protocol individually and combinatorically tested gradient, shift, and drive fields. The table indicates the number of healthy subjects that reported sensations of magnetostimulation in the foot or head for each set of fields. Only one subject (S04) experienced magnetostimulation during tests involving the drive field. **(f)** S04’s magnetostimulation results are plotted and fit to a logistic regression to determine the 50% probability, 50%−1*σ* and 50%−2*σ* amplitudes. Since no gross differences in stimulation thresholds were experienced from the drive field in combination with additional fields or preversus post-injection, all of S04’s drive field magnetostimulation results are pooled. The results are used to determine the maximum drive field amplitude for use in this subject’s image sequences.

Our clinical imager has a 25 cm imaging bore to fit the head and neck or extremities, and a field of view (FOV) of 24 × 24 × 30 cm to enable imaging from crown to laryngeal prominence (Extended Data Figure 2,3,5). The design utilized a multi-receive-coil architecture combining volume and surface coil arrays (Extended Data Figure 5,6). The application-specific surface coil arrays enable improved sensitivity and shine-through performance, and in this study are positioned close to the head and neck near the anatomical location of expected lymphatic drainage pathways. Prior to imaging, the imager was verified for basic safety and adherence to relevant FDA risk guidance for MRI (see Methods). We simulated and experimentally verified specific absorption rate (SAR), and tested magnetostimulation thresholds for peripheral nerve stimulation (PNS) and magnetophosphene perception (Figure 3). SAR was simulated for a human body model and a cylindrical phantom, and the phantom results were experimentally verified, which confirmed SAR to be below the threshold used in MRI for significant risk operating conditions (see Methods, Figure 3a,b). Thresholds for magnetostimulation were simulated up to 7 mT peak drive field (Figure 3c) and then experimentally tested up to the system’s designed maximum drive field strength of 3.5 mT peak (Figure 3d,e) in 6 healthy subjects (4 heads, 6 feet) and again after magnetic tracer injection in 2 subjects (2 heads, 2 feet). In the head, one subject (n=1/4) experienced threshold magnetostimulation (PNS) at a drive field amplitude of 3.2 mT peak (50% probability), which occurred near an MRI compatible gold-alloy dental crown. In the foot, no subjects (n=0/6) experienced magnetostimulation. There was no detectable change in magnetostimulation threshold after magnetic tracer injection.

**Figure 4:**
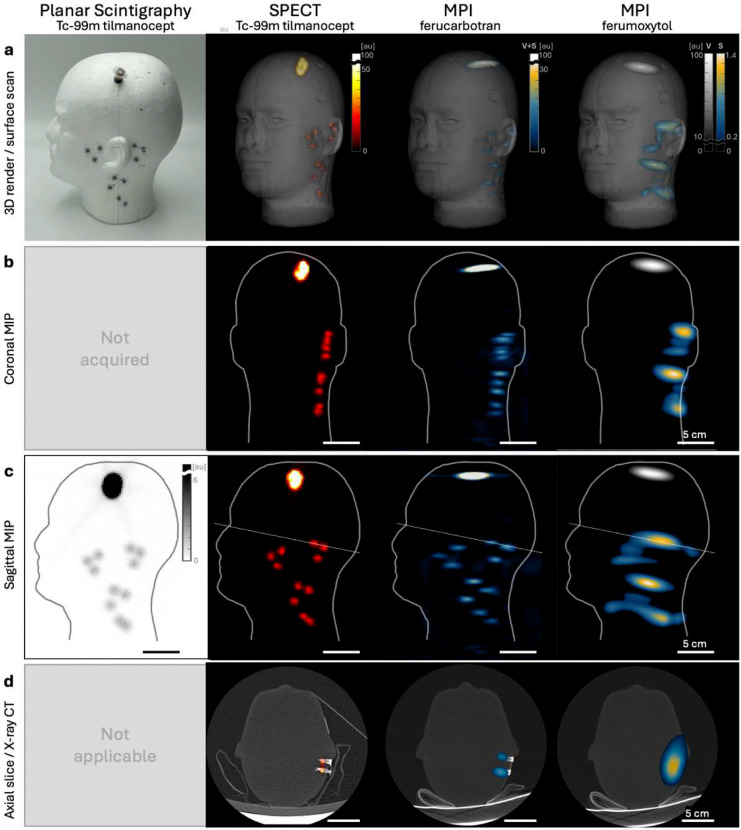
Clinical performance benchmarking of MPI against standard-of-care nuclear imaging. A lymph node head-and-neck phantom containing a 1.5 mL Eppendorf tube in the parietal scalp and 11× 0.2 mL Eppendorf tubes in the periauricular and cervical lymph node regions. The phantom was filled with a 100:1 tracer quantity difference between mock injection and lymph nodes, with [^99m^Tc]tilmanocept for planar scintigraphy and SPECT or with ferucarbotran or ferumoxytol for MPI. Scale bars represent 5 cm. **(a)** Photograph of phantom, and 3D volume rendering of SPECT and MPI images co-registered with an optical surface scan. **(b)** Coronal maximum intensity projection (MIP) of SPECT and MPI (not acquired for planar scintigraphy). **(c)** Sagittal MIPs of SPECT and MPI, and sagittal projection of planar scintigraphy. **(d)** Oblique axial slice of SPECT/CT and MPI/CT at the dotted-line drawn in (c) (not applicable for planar scintigraphy).

To benchmark MPI performance against standard clinical lymphatic imaging methods, we imaged anthropomorphic phantoms with MPI, planar scintigraphy, and SPECT (Figure 4). Two magnetic tracers were evaluated: ferumoxytol and ferucarbotran. With ferucarbotran, we observed image resolution comparable to [^99m^Tc]tilmanocept SPECT, with MPI showing similar axial resolution (z) but lower transverse resolution (x,y). With ferumoxytol, MPI showed markedly worse resolution, as confirmed in phantoms (Extended Data Figure 4).

**Figure 5:**
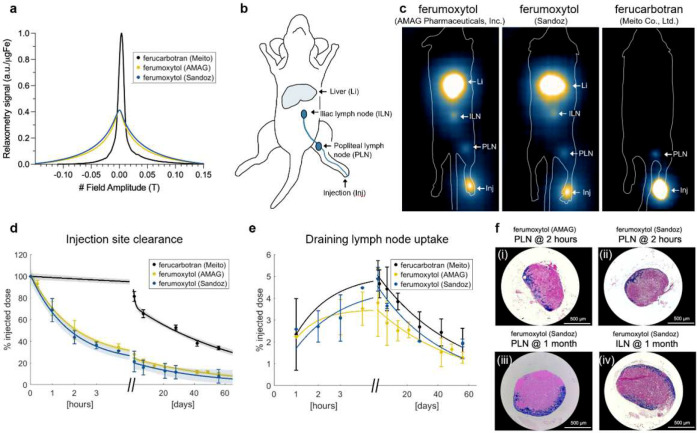
Tracer properties drive magnetic and pharmacokinetic performance, demonstrated in an *in vivo* mouse model with histological confirmation. **(a)** MPI relaxometry measures the signal strength (maximum) per μg Fe, and resolution (full width at half maximum) for each tracer. Ferumoxytol shows ~40% lower signal strength and 5–6× worse resolution compared to ferucabotran, with minimal differences between ferumoxytol manufacturers. **(b)** Mouse lymphatic anatomy showing footpad injection site (Inj) and expected draining nodes^37^. **(c)** Full-body mouse MPI shows biodistribution of ferumoxytol (AMAG Pharmaceuticals) (n=7), ferumoxytol (Sandoz) (n=3), and ferucarbotran (Meito Co., Ltd.) (n=3), 24 hours after tracer injection to the mouse footpad. Magnetic tracers are detected in the draining popliteal lymph node (PLN), secondary iliac lymph nodes (ILN), and liver (Li). **(d,e)** Longitudinal quantification of MPI signal, where time-series data are fit to a bi-exponential (solid lines) with 95% prediction intervals (shaded region), shows **(d)** rapid clearance of ferumoxytol (AMAG and Sandoz) from the injection site and **(e)** accumulation in lymph nodes within 4 hours and for 8 weeks. Comparatively, ferucarbotran shows slower clearance kinetics and accumulation in lymph nodes was detected by MPI after 24 hours. **(f)** Perl’s Prussian blue staining confirms iron staining following ferumoxytol injection, in excised draining PLN at (i) 2 hours after AMAG product, (ii) 2 hours after Sandoz product, (iii) 1 month after AMAG product, and (iv) in the ILN at 1 month after Sandoz product. Scale bars = 500 μm.

To further explore lymphatic imaging with MPI and compare tracer-dependent pharmacokinetics in vivo, we performed studies in healthy mice using ferumoxytol and ferucarbotran (Figure 5). MPI showed a rapid clearance of ferumoxytol from the injection site and uptake into multiple lymph node echelons before accumulating in the liver (Figure 5c,d). MPI signal in lymph nodes was detected as early as 1 hour after injection and persisted for at least 8 weeks (Figure 5e), and iron deposits were confirmed by *ex vivo* Prussian blue staining (Figure 5f). Ferumoxytol sold by AMAG Pharmaceuticals (n=7) and Sandoz (n=3) showed similar magnetic properties and lymph node deposition (Figure 5a,f). Ferucarbotran (n=6) exhibited slower clearance from the injection site and was detected in only the first draining sentinel lymph node, consistent with the reported pharmacokinetics of Magtrace^34–36^.

**Extended Data Figure 8:**
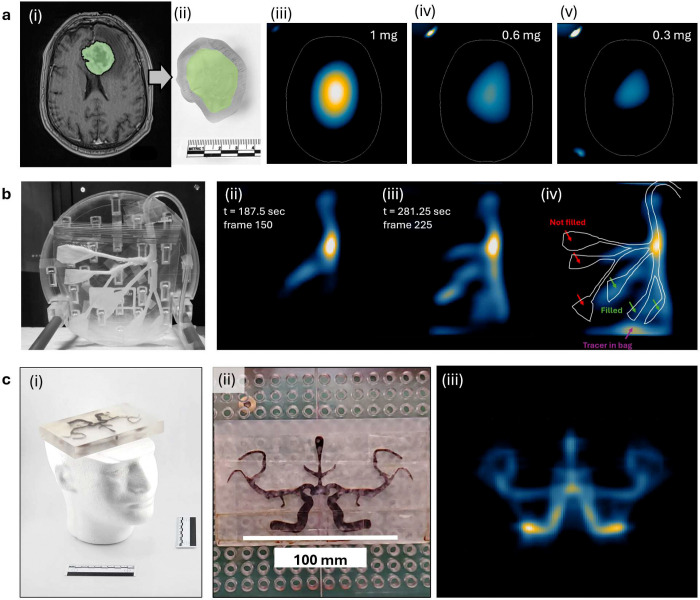
MPI visualization of phantoms simulating brain tumor imaging, real-time embolization, and tomographic angiography at clinical-scale. **(a)** Proof-of-concept MPI detection of a human-derived glioblastoma tumor phantom filled with ferumoxytol. (i) A glioblastoma volume was segmented from a patient brain MRI (UPENN-GBM collection) and used to produce (ii) a 3D printed, hollow, fillable tumor phantom. (iii,iv,v) MPI scans of the phantom filled with ferumoxytol at (iii) 1.0 mg Fe (iv) 0.6 mg Fe and (v) 0.3 mg Fe, represent 0.2%, 0.12% and 0.06% of approved dose 510 mg, respectively. MPI demonstrates concentration-dependent signal at human scale. **(b)** Real-time 2D axial slice imaging at 0.8 frames/second of a trans-arterial embolization liver phantom being filled with ferucarbotran (see also **Supplemental Video SV2**). (i) Photo of the phantom prior to filling with tracer. (ii,iii) Frame grabs from real-time imaging of the phantom filling with tracer. Each image is a 2D slice, 20 × 20 cm. (iv) Final frame taken after filling that shows multiple branches did not fill with tracer. **(c)** (i,ii) Photos of phantom modeling arterial anatomy based on a 2D MRI image, filled with ferucarbotran. Approximate human-sized Styrofoam head for scale. (iii) A two-scan MPI image was taken, with rotation of the phantom by 90 degrees between scans to simulate the use of a multi-axis drive field. The two scans were aligned and summed to yield the final image.

The MPI hardware developed here has applications beyond clinical lymphatic imaging, outside the scope of our human study. In anticipation of future uses, we explored imaging phantom models of brain cancer, real-time embolization, and cerebral angiography (Extended Data Figure 8, **Supplemental Video SV2**).

## Discussion

First-in-human images with MPI represent a milestone in medical imaging, demonstrating translation of an emerging tracer imaging modality to clinical research. Until now, no images of human subjects have been reported^28^. We showed longitudinal and quantitative visualization of magnetic tracer pharmacokinetics in humans, with sufficient sensitivity and resolution for clinical applications that can benefit from these capabilities such as presurgical imaging for sentinel lymph node biopsies. These milestones were reached safely in healthy subjects. MPI’s unique technical characteristics open new applications benefiting from hotspot image contrast, weeks-long signal half-life, shelf-stable tracers, and no ionizing radiation.

MPI has evolved over two decades; preclinical MPI is commercially available^38^, and several human-scale prototype scanners have been developed^39–44^. These scanners have imaged non-human primates^19,20^ and human cadaver leg specimens^21^, but until now, no images of human subjects have been reported. To achieve human MPI images, we constructed novel hardware, and designed and verified it following a risk-based approach for use in an Institutional Review Board approved study. The hardware is sized for head-and-neck (field of view of 24 × 24 × 30 cm), and achieves the sensitivity, dynamic range, and shine-through performance sufficient for lymph node imaging applications.

Our study established MPI’s clinical feasibility by demonstrating lymphatic imaging, where MPI can fill gaps in current clinical workflows and capabilities. The most used lymphatic imaging technique, the sentinel lymph node biopsy (SLNB) guided by ^99m^Tc radiotracer-based imaging, is widely used for staging early-stage breast cancer, melanoma, and head-and-neck malignancies^4,6,32,45^. The short half-life of ^99m^Tc (6 hours) necessitates same or next day coordination of tracer injection, presurgical imaging, and surgery^4^. Emerging magnetic SLNB methods using iron oxide nanoparticles and handheld magnetic probes eliminate this timing constraint, allowing at least 7 days between injection and surgery^46^, but lack presurgical imaging capability and are limited to use in patients with breast cancer^35^.

Here we demonstrate MPI lymphatic visualization, with the goal of supporting presurgical imaging for magnetic SLNB workflows in melanoma and head and neck cancer, where imaging remains essential for surgical planning as drainage patterns are complex and variable^4,32^. The human MPI scanner achieved sufficient sensitivity, dynamic range, and shine-through performance to visualize magnetic tracer pharmacokinetics in the lymphatic system with no background signal arising from tissues (Figure 1). We acquired MPI images of two subjects with subcutaneously injected magnetic tracer in parietal scalp, and detected MPI signal in periauricular and cervical lymph node regions (Extended Data Figure 7), consistent with prior MR lymphangiography studies performed by the authors utilizing ferumoxytol (unpublished data). Quantification of magnetic tracer was performed in humans for up to 28 weeks (Figure 2). We then tested image quality in phantoms across modalities and tracers, and found that MPI with ferucarbotran, a tracer shown to have suitable axillary sentinel lymph accumulation for intraoperative surgical guidance using a handheld detector^47^, has comparable image quality to planar gamma camera and SPECT with [^99m^Tc]tilmanocept at clinically relevant tracer quantities (Figure 4). These findings are supported by preclinical mouse studies (Figure 5), which establish biological validation, cohort-level robustness, and scalability of lymphatic MPI from mice to humans.

Although clinical MPI systems and tracers are in their first generation, in Figure 4 we benchmark MPI against state-of-the-art SPECT and planar scintigraphy. The phantoms were designed to represent and approximate lymph node anatomy draining from a melanoma site in the parietal scalp^31–33^. The magnetic and radiotracer phantom were both filled with a 100:1 tracer quantity ratio between a mock injection site and draining lymph nodes, modeling approved tracer doses^35,48^ and reported deposition in lymph nodes^49–51^ (Figure 4). The imaging results highlight the importance of tracer selection on imaging performance. With ferucarbotran, MPI achieves comparable image quality to SPECT with [^99m^Tc]tilmanocept, with similar per node detectability. On the other hand, ferumoxytol, the tracer used in our human subjects, showed approximately 5-fold worse spatial resolution than ferucarbotran on identical hardware (Extended Data Figure 4,5a). This difference arises because MPI spatial resolution depends on the magnetic nanoparticle’s magnetization curve steepness in addition to hardware specifications^1^. Compared to ferumoxytol, ferucarbotran has larger magnetic core size which produces steeper magnetization transitions and correspondingly better image quality^2,13^. Our phantom data establish that future MPI studies using ferucarbotran or purpose-designed tracers should achieve substantially improved spatial resolution without changing hardware specifications^52,53^. As MPI hardware and tracers mature, we expect imaging performance to improve in field of view, sensitivity, resolution, and dynamic range, similar to how MRI, CT, and nuclear medicine evolved rapidly in the 1970s and 1980s.

Our preclinical studies (Figure 5) were integral to the design of the human imaging protocol and provided independent biological validation of human lymphatic imaging. Mouse data informed study design by confirming ferumoxytol visibility with MPI, establishing expected pharmacokinetic behavior with whole-body context, and guiding selection of imaging time points. In mice, ferumoxytol reproducibly cleared rapidly from the injection site and accumulated in multiple lymph node echelons within hours, with quantitative signal stability persisting for at least 8 weeks, consistent with trends observed in human subjects. Notably, the circulation dependent decay rate *τ*_1_ has a ~10-fold difference between mouse and human, which is consistent with the expected allometric scaling of circulation time (M^0.25)^54^ (Extended Data Table 2). The mouse model enabled direct validation of imaging findings through excision of lymph nodes and histological confirmation of iron deposition, providing biological ground truth that is not feasible in human studies. Mouse imaging also supported the interchangeability of brand-name and generic ferumoxytol used in the human study. Finally, it enabled a direct comparison of the pharmacokinetic behavior of ferumoxytol and ferucarbotran, which have different coatings and hydrodynamic diameters (17–31 nm^29^ and ~57 nm^47^, respectively), highlighting tracer-dependent pharmacokinetic variability and informing future tracer selection. While we did not inject ferucarbotran into human subjects, these preclinical studies reaffirm that it is suitable as a sentinel lymph node magnetic tracer^3,49^, accumulating primarily in the first nodal echelon. Together, the strong concordance between mouse and human results strengthens confidence in translating MPI from small-animal models to broader clinical applications.

Human translation required a novel main magnet. In order to achieve an axial FOV from the crown to the laryngeal prominence, the system was designed with a 55 cm free bore to fit the shoulders and torso, allowing shifting of the subject through the isocenter (Extended Data Figure 2,3). This main magnet design can scale to whole-body imaging systems, with larger bores and extended axial coverage while maintaining a field gradient greater than 0.5 T/m.

Many clinical applications, including lymphatic imaging, benefit from strong shine-through performance, which is the ability to resolve small signal sources nearby a large source. We improved shine-through performance using a unique combination of volume and surface receive coils, where multi-channel volume coils enable large fields of view with uniform receive profiles and application-specific surface coils provide localized high sensitivity (Extended Data Figure 5,6). We note that shine-through performance relies on having sufficient imager dynamic range, which is the ratio of the largest to smallest signals that can be imaged in isolation, and can span 4–6 orders of magnitude before detector saturation occurs. In our study, the application-specific surface coil arrays were placed in close proximity to the expected lymphatic drainage basin, both improving dynamic range and reducing shine-through artifact when visualizing milligram-level injection sites next to microgram-level lymph node accumulation. These coils are used throughout our human study, and when used with ferucarbotran in our SLN phantom can visualize an 8000:1 tracer ratio between injection site and lymph nodes, which exceeds expected physiologic dynamic range by at least an order of magnitude (Extended Data Figure 6d).

Our study had several limitations. Our testing with ferumoxytol did not demonstrate human imaging at MPI’s full imaging performance capabilities, and future clinical tests using optimized tracers are expected to achieve substantially better image quality than demonstrated here (Figure 4, MPI ferucarbotran versus MPI ferumoxytol). While Magtrace, an FDA-approved medical device for SLNB procedures^35^ has performance similar to ferucarbotran (data not shown), it was not available for our study; ferumoxytol was readily available and was used off-label from its FDA-approved indications as an iron replacement therapy and for cancer neuroimaging^29,30^. As a healthy subject study, *in vivo* imaging comparisons with existing clinical modalities was not justified, particularly those necessitating radioactive tracer administration. We instead relied on phantoms to compare relative performance of MPI with scintigraphy and SPECT (Figure 4). In future MPI studies in patient populations, dual tracer techniques may be warranted and would enable magnetic versus nuclear imaging comparisons. Nodal biopsy in healthy subjects was also not justified, and so to confirm localization of MPI signal in lymph node regions, we relied on imaging experiments that show tracer signal follows anatomy when repositioning the subject or receiver coils (Extended Data Figure 7). Our small sample size (n=4 experiments with tracer injection, n=10 experiments for magnetostimulation threshold testing) provided limited statistical power for assessing inter-subject variability, though consistency between subjects and agreement with mouse data suggest reproducibility. The magnetostimulation threshold testing served its primary purpose to set subject-specific parameters for imaging sequences, but as it was only performed up to 3.5 mT peak and only achieved thresholds in one subject (at a location of prior dental work), it does not provide generalizable results. Last, the imager’s field of view restricted imaging to head and neck anatomy, precluding assessment of lymph node regions inferior to the laryngeal prominence. We also were unable to image the extent of the foot lymphatic basin including popliteal or inguinal lymph nodes^55^ that we were unable to position within the imaging region.

MPI addresses an unmet need in tracer imaging beyond lymphatic imaging with radiation-free, quantitative, sensitive, and sustained measurements. With existing tracers approved for human use and the scanner technology developed here, MPI may already be able to supplement or replace MRI, X-ray, and radiotracer-based studies. For example, ferumoxytol recently received FDA approval for brain tumor imaging with MRI^30^ (Ferabright^™^, Azurity Pharmaceuticals), and MPI could provide complementary directly quantitative measurements of tracer present in the tumor^13^. We demonstrate feasibility for human-scale tumor imaging with ferumoxytol by imaging a glioblastoma phantom filled with tracer at 0.06% of the approved dose^30^ (Extended Data Figure 8a). MPI may also find use in monitoring interventional procedures^21,44^, without the limitations of radiation dose and tracer toxicity, such as transarterial embolization procedures and angiography, traditionally performed using X-ray-based technologies including fluorscopy and X-ray CT. To model these interventional procedures, we performed real-time imaging of an arterial phantom filling with tracer, and static, tomographic imaging of an anigiography phantom (Extended Data Figure 8b,c, **Supplemental Video SV2**). MPI can also address new applications such as monitoring cell therapies^7–12^ or imaging metastasis-associated inflammation^13,14^, especially paired with the ongoing development of magnetic tracers designed with application-specific coatings and improved MPI performance^52,53^. Recent work has also shown MPI can detect the magnetic signal from hemosiderin, an endogenous hemoglobin degradation product, and was demonstrated as a high-contrast biomarker for unstable plaques by imaging intraplaque hemorrhage in human endoarterectomy samples and an *in vivo* animal model^22^.

In this work, we report the first-in-human imaging with MPI in healthy subjects and establish the clinical feasibility of this technology. MPI introduces a unique imaging physics principle to tomographic medical imaging, comparable to the introduction of clinical gamma camera, PET, Ultrasound, MRI, and CT. Until now, MPI capabilities had only been demonstrated in small animals but lacked a pathway to testing in humans. This work demonstrates that pathway, enabling the exploration of MPI across a broad range of clinical applications, and providing a foundation for future studies in patients.

## Methods

### MPI Clinical Imager.

An MPI image is acquired by moving a field-free region throughout a field of view, applying a drive field, and measuring signals induced in receive coils by magnetic tracers. The saturation of the tracer’s magnetic response in a strong field enables spatial encoding of signals near the instantaneous position of the field free region^25,56,57^ (Extended Data Figure 1). In this work, a field free point (FFP) is generated by a main magnet and shifted both magnetically and mechanically to cover the field of view. A drive assembly produces a pure sinusoidal field only within the field of view, and volume and surface receive coils detect the signal produced by the tracers. These subsystems are controlled using a realtime controller (National Instruments, Austin, TX) and all imaging is performed in a magnetically shielded room (ETS-Lindgren, Cedar Park, TX). Complete system specifications are provided in Extended Data Table 1.

The main magnet produces a strong gradient field containing a single point where the total magnetic field passes through zero, the FFP, and electronically shifts the FFP across a FOV (Extended Data Figure 2,3). The main magnet is constructed using four electromagnets arranged in a quadrupolar configuration on an iron yoke. The gradient is generated by applying dc currents, and transverse (x, y) shifting is performed by superimposing additional time-varying currents. The gradient strength at the FFP is 0.3 × 0.3 × 0.6 T/m, and typical shifting fields of +/− 36 mT allow sampling across an axial (x/y) slab up to 24 cm × 24 cm. The magnet is constructed using water-cooled hollow conductors, driven using current-controlled amplifiers (IECO Oy, Helsinki, Finland), and dissipates ~80 kW during operation. The FOV is sampled axially (z) in discrete slabs by mechanically stepping the subject bed position across a 30 cm range.

The drive field interacts with the magnetic tracer to produce a signal. The field is a homogenous, axially oriented (z), sinusoidally varying magnetic field at a frequency of 45.03 kHz and an amplitude of up to 3.5 mT peak at the isocenter (Extended Data Figure 2,5). The drive field (referred to interchangeably as “transmit”) is produced by a water-cooled resonant solenoid coil and capacitor bank (Celem Capacitors, Jerusalem, Israel) mounted to the subject bed. The coil is powered by an industrial audio-frequency amplifier (8504, AE Techron, Elkhart, IN) whose output is filtered with a balanced, fourth order passive filter. The drive field signal chain dissipates up to 875 W, with 550 W dissipated into the transmit coil, 50 W dissipated in the bore due to induced eddy currents, and 275 W into the filter. During operation, active feedback control maintains transmit phase and amplitude.

Volume and surface receive coils inductively detect signals from magnetic tracers within the FOV. A pair of volume coils (Extended Data Figure 5), whose winding patterns are inspired by Hadamard encoding, are mounted concentrically inside the drive coil. The first receive coil is a 2-part (+1, −1) gradiometer, and the second receive coil is a 3-part gradiometer (+1/2, −1, +1/2), each wound to minimize feedthrough from the transmitter. Each gradiometer part has a typical operating voltage of up to ~1.4 kVp, and the sum of the parts cancels the voltage at the coil output to a manageable <10 Vp. While each volume coil individually has null regions within the field of view, the coils are designed with complementary sensitivity patterns so that when combined during reconstruction, they provide uniform signal coverage across the field of view without sensitivity null regions. In contrast to the uniform sensitivity of the volume coils, the sensitivity of the surface coils varies spatially and drops off with distance from the coil’s surface, which can be used to selectively detect signals from a desired region of interest. Surface coils (Extended Data Figure 6) are mounted to a rigid application-specific head cradle positioned inside the volume coils. The cradle accurately locates up to four surface coils next to the cervical and auricular lymph node regions. Each surface coil conductor is constructed as a printed circuit board and designed with a conductor winding optimized for high sensitivity at distances of 1.5–3 cm from the coil surface while minimizing coupling to the volume transmit coil. Human data presented utilized a left-side only coil configuration, as magnetic tracer injections were in the left parietal scalp and subjects showed unilateral lymphatic drainage. The voltages from the 4 receive channels, 2 volume and 2 surface, are filtered with a balanced-input passive notch filter (133 dB attenuation) to remove remaining feedthrough from the transmitter prior to amplification with a custom low-noise preamplifier with a differential input based on the JFE2140 (Texas Instruments, Dallas, Texas, USA).

An imaging pulse sequence acquires a 3D volume up to 24 × 24 × 30 cm by acquiring axial slabs with mechanical bed shifts between slabs (Extended Data Figure 2c). For each slab, a raster acquisition sequence applies a magnetic field gradient, shifts the FFP position electronically across x and y using a triangular waveform, and applies a drive field along z. A slab acquisition covers FOVxy = 20–24 cm, typically with y-direction fast shift at 4 traversals per second and an electronic raster step size of dx = 5 mm. Axial coverage (FOVz) is typically 10–28 cm with mechanical step size of dz = 5 mm to provide sufficient spatial encoding^58^. For FOVz = 28 cm, total scan time is approximately 15 minutes. Sequence parameters for all MPI images taken on the clinical scanner are reported in Supplementary Table ST1.

### Magnetic Tracers.

Phantom, preclinical, and clinical studies utilized ferumoxytol and the active ingredient in ferucarbotran, dextran magnetite (Meito Co. Ltd., Nagoya, Japan), which we refer to as ferucarbotran in this document. Ferumoxytol is an iron oxide coated with polyglucose sorbitol carboxymethylether with a 17–31 nm hydrodynamic diameter^29^, and is approved for iron replacement therapy^29^ and cancer neuroimaging^30^. Ferumoxytol was obtained from two manufacturers, AMAG Pharmaceuticals (Feraheme; Waltham, MA, USA) and Sandoz (generic; Basel, Switzerland). Ferucarbotran is an iron oxide coated with carboxydextran with a ~57 nm hydrodynamic diameter^47^, and is approved for human use as an MRI contrast agent in Germany and Sweden (Resotran; B.E. Imaging GmbH, Baden-Baden, Germany)^59^ and Japan (Resovist; PDR Pharma Co. Ltd., Tokyo, Japan)^60^, and was not available for our study. Magtrace, an FDA-approved medical device for SLNB procedures^35^ with MPI imaging performance comparable to ferucarbotran (data not shown), was also not available for our study.

### Signal Processing & Image Reconstruction.

The analog signals are digitized at 4 MSPS by the realtime controller and digitally filtered to isolate the 90–250 kHz frequency band. The time domain signals are transformed into harmonic portraits dk(x,y,z) (where k=harmonic number), by filtering harmonic bands of the drive field frequency and gridding them to the instantaneous FFP position (Extended Data Figure 1b). The harmonic portraits are reconstructed into volume or surface coil specific images using multi-harmonic gridded 3D deconvolution (MH3D)^58^, which is a convolutional forward model that incorporates the transmit and receive coil sensitivity patterns and the magnetic tracer’s point spread function.

For each scan, volume coil images and, when applicable, surface coil images are reconstructed. When both are used, the images are typically combined using coil sensitivity-weighting into a single composite image displayed in a perceptually linear false color colormap designed for MPI (“bluesteel”, black-blue-gold, Supplemental Figure S2, Supplemental Table ST2). However, in the case of ferumoxytol at high dynamic range, low native resolution and low sensitivity worsen shine-through artifact and reconstruction noise, making a single composite visualization challenging. Therefore, to better visualize the surface coil signal with this tracer, we display the volume coil data in grayscale and the surface coil data in bluesteel as separate overlays rather than compositing them into a single image. In all cases, resulting MPI images are displayed either as maximum intensity projections (MIPs) created using MATLAB (2024b, MathWorks, Natick MA) or as volume renderings co-registered with an optical scan or X-ray CT using 3D Slicer^61^.

### Basic Safety.

The imager was verified using a risk-based approach against a subset of requirements defined by the IEC and FDA for MRI, in this work defined as “basic safety” testing. Our verifications included general requirements for medical equipment^62^, with a focus on electrical safety. For MPI-specific verifications arising from the use of time-varying magnetic fields^63^, we evaluated the imager against MRI guidance for Specific Absorption Rate (SAR) in order to support Nonsignificant Risk determination and performed magnetostimulation studies to determine stimulation thresholds in human heads and feet^64–66^. We detail the SAR and magnetostimulation testing below.

### Specific Absorption Rate (SAR) Simulation and Phantom Testing.

SAR was simulated on an anatomically realistic virtual human body model (“Duke”) and a saline phantom using Sim4Life (ZMT Zurich MedTech AG) and experimentally verified for the phantom (Figure 3). See Supplementary Methods for details.

### Human PNS Threshold Simulation.

Peripheral nerve stimulation (PNS) is the activation of nerves in response to a time-varying magnetic field. Electromagnetic simulations were performed to determine the onset of PNS in a virtual body model for the drive field conductor up to 7 mT peak. The simulation used the Sim4Life software and estimated the onset of PNS using the drive field conductor model used in the SAR simulations, the “Yoon-sun” virtual human body models, and the NEURON Solver implementing the “McIntyre-Richardson-Grill” (MRG) neuronal model.

### Human Magnetostimulation Threshold Testing.

The magnetostimulation threshold studies were designed to measure the onset of PNS and/or magnetophosphenes, primarily informing the selection of imaging sequence parameters. When experienced by a subject, the PNS stimulation at threshold is experienced as a perceptible activation of peripheral nerves such as light tingling or twitching, and magnetophenes at threshold are experienced as faint flickering visual sensations^64^. Prior experimental MPI human PNS testing focused on the drive fields^67–69^. Here we test all magnetic fields used in an imaging sequence. The study was approved by the Advarra (Protocol #Pro00071449) Institutional Review Board and the Stanford Institutional Review Board via reliance.

Magnetostimulation thresholds were measured in 6 healthy adult subjects (n=6 feet, n=4 head). The study used a systematic protocol to isolate effects of each magnetic field component for the gradient, shift, drive field, as well as combinations of all the fields. For each configuration, field amplitude was first increased in coarse steps up to the maximum amplitude. During testing, subjects reported any sensations or visual perceptions. In the event of stimulation, the process is then repeated with finer step sizes starting below the coarse threshold. Last, the subject is tested with random amplitudes within ±15% of the fine threshold. Because higher frequencies (e.g., >10 kHz) tend not to have a sharp transition at the stimulation threshold, this fine sampling and statistical modeling approach enables accurate and consistent measurement of the amplitude at which a subject has a 50% probability of stimulation^67^. To determine this 50% probability amplitude, the binary magnetostimulation data was fit to a logistic regression (Figure 3f).

Study demographics and results are shown in Extended Data Table 2 and Figure 3. For subjects that experienced magnetostimulation, results were used to set field amplitudes for subsequent imaging experiments.

### Human Imaging with Tracer Injection.

A subset of subjects enrolled in optional human imaging with tracer injection substudies. The substudy enrolled 2 healthy adult subjects (n=2 feet, n=2 head). The study was approved by the Advarra (Protocol #Pro00071449) Institutional Review Board and the Stanford Institutional Review Board via reliance. Adverse events were classified using the Common Terminology Criteria for Adverse Events (CTCAE) v5.0^70^; if the adverse event was not specifically listed in the CTCAE, the general grading guidelines were applied.

For the head injections (n=2), subcutaneous injections were performed in the parietal scalp. For the foot injections (n=2), subcutaneous injections were performed in the lateral heel, between the lateral malleolus and Achilles tendon. The tracer injected in all cases was ferumoxytol, manufactured by either AMAG Pharmaceuticals or Sandoz (see table below). All dilutions were performed immediately prior to injection in a 1:1 ratio with sterile saline. All injections were performed by a physician at Stanford University Medical School, Stanford, California, USA.

Injection details by anatomy and subject:

**Table:** 

Anatomy	Subject	Ferumoxytol Source	Iron Quantity	Injection Volume
Head	S05	AMAG Pharma.	7.5 mg	0.5 mL
Head	S04	Sandoz	15.0 mg	1.0 mL
Foot	S04	AMAG Pharma.	7.5 mg	0.5 mL
Foot	S05	AMAG Pharma.	15.0 mg	1.0 mL

Following injection, the subject was monitored for 30 minutes, then transported to the study site and first underwent repeat magnetostimulation protocol, resulting in a ~2 hr delay before the first imaging timepoint.

Longitudinal imaging was performed via multiple imaging sessions post-injection. To capture expected tracer clearance rates, imaging sessions occurred more frequently in the week following injection (day 1–3, etc.) and then more widely spaced through the experimental window (1 week–6 months). While consistent sequence parameters were targeted for longitudinal imaging of injection sites, the exploratory nature of the lymphatic imaging resulted in some variation in sequence parameters. Sequence parameters for all images used in the analysis are specified in Supplementary Table ST1.

The injection site was photographed after each imaging session to monitor skin discoloration. Subjects were scanned with a 3D camera (Einstar Vega, Shining 3D, Hangzhou, China). MPI and optical scans were co-registered using MPI/optically-visible fiducials in 3D Slicer^61^.

### Human Data Image Analysis & Quantitative Calibration.

Injection sites and lymphatic regions were analyzed using region-specific image processing workflows. For the injection site, signal was quantified over time and calibrated to quantify magnetic tracer quantity over time. For the lymphatic region, signal was quantified over time but not calibrated, due to higher signal variability caused by scan-to-scan changes in subject positioning and in imaging sequence parameters, complicated by the field nonuniformity in the surface coils.

Injection site signal quantification was performed by integrating the signal over a 3D rectangular prism ROIInj (10 × 10 × 4 cm) of the volume coil reconstruction (V) for each image in the time series (Figure 2a).

Injection site signal calibration established an integrated signal-to-tracer quantity scaling factor using a phantom modeling the injection site. The phantom was constructed by loading a 2.25 cm disc-shaped cotton absorbent pad with tracer matching the subject’s injection quantity and volume. The pad was used to mimic the distribution of tracer following subcutaneous injections. It was placed in a resealable plastic sleeve, and affixed to a head phantom at the approximate injection site location for imaging using the same sequence and parameters.

Head-and-neck lymph node region signal quantification was performed by integrating the signal over a 3D rectangular prism ROILNs of the surface coil reconstruction (S) for each image in the time series (Figure 2a). Only the surface coil reconstruction was used, as the volume coils lacked sufficient sensitivity and shine-through performance for imaging ferumoxytol in the lymph nodes. With ferumoxytol’s low spatial resolution, individual lymph nodes were not expected to be resolvable, so signal was integrated over the entire region rather than reported for individual nodes. The ROILNs dimensions (S04: 14 × 14 × 12 cm, S05: 12 × 14 × 10 cm) were set for each subject, and consistent across all longitudinal time points (Supplemental Table ST1).

### Time-Series Data Analysis.

Time-series analysis was performed by fitting parametric models in MAT-LAB (2024b, MathWorks, Natick MA). The injection site was modeled assuming monotonic tracer clearance. Lymphatic uptake and clearance were modeled assuming signal accumulation and decay occur concurrently. Best-fit parameter estimates are reported in Extended Data Table 2b. The time-series analyses were performed independently for the injection site and lymph node regions. Sequence parameters for all images used in each analysis are listed by figure in Supplemental Data Table ST1.

Injection site clearance dynamics were modeled by a bi-exponential decay, and the decay time values are reported for a least-squares fit to measured data using a solver built on nonlinear Nelder-Mead optimization (*fminsearch*). Prediction intervals (95%) were estimated using a parametric bootstrap with Student-t noise and a small-sample variance correction to avoid undercoverage given limited data points^71^. The images used in the injection site time-series analysis were acquired using a predefined imaging sequence and subject position.

Lymphatic update and clearance dynamics were modeled by a two-pole overdamped model, which can be shown equivalent to a two-compartment model^72^, and the time constants are reported for a least-squares fit to measured data. Parameters and their 95% prediction intervals were estimated by nonlinear least-squares regression (*fitnlm*). The images used in the lymphatic time-series analysis pooled a few groupings (detailed in Supplementary Table ST1) of imaging sequence parameters and head rotation positions (positions illustrated in Extended Data Figure 7a). The pooling balanced time-course sampling density with the high image-to-image parameter variability. Error bars in Figure 2d show the average and standard deviation for images grouped by same acquisition day.

### Imaging Performance Characterization.

The system performance was characterized for spatial resolution, shine-through, and sensitivity using phantoms with both ferumoxytol and ferucarbotran (see Extended Data Figures 4-6 and Supplementary Methods for details).

### Imaging Performance Benchmarking with SPECT and Gamma Camera.

Phantom studies were completed to benchmark MPI image quality against Tc-99m scintigraphy and SPECT in head and neck lymphatic imaging. A phantom was constructed from a Styrofoam mannequin head (SmoothFōM^®^ Male Foam Head, FloraCraft^®^, Ludington, Michigan) with holes for a 1.5 mL tube in the left parietal scalp to model the injection site, and eleven 0.2 mL tubes in periauricular and cervical regions to model draining lymph nodes on the same (left). Images were acquired on the phantoms using planar scintigraphy, SPECT, MPI with ferucarbotran and MPI with ferumoxytol. See Supplementary Methods for details.

### Preclinical Animal Testing & Tracer Relaxometry.

Mouse tracer pharmacokinetics studies were conducted at the Robarts Research Institute under an approved protocol from Western University’s Institutional Animal Care and Use Committee (Protocol #2023–113) and adhered to ARRIVE guidelines.

Mice received subcutaneous hind-footpad injections of one of three tracers: ferumoxytol manufactured by AMAG Pharmaceuticals (50 μg, n=7), ferumoxytol manufactured by Sandoz (50 μg, n=3), or ferucarbotran (25 μg, n=6) in a volume of 25 μL.

MPI images were acquired on a preclinical MPI scanner (Momentum, Magnetic Insight Inc., Alameda, CA) at multiple time points up to 8 weeks post-injection to quantify magnetic tracer pharmacokinetics.

At study endpoint, mice were euthanized and the popliteal and iliac lymph nodes were excised and processed for Perl’s Prussian blue staining to confirm iron deposition.

Magnetic properties of ferumoxytol (AMAG Pharmaceuticals vs. Sandoz) were characterized using magnetic particle relaxometry (RELAX module, Momentum™, Magnetic Insight Inc, Alameda, CA).

## Extended Data

**Extended Data Table 1:** System specifications and measured performance.

Parameter	Measured Performance	Notes
Magnet bore diameter	60 cm	Pole piece to pole piece
Patient bore diameter	55 cm	Inside copper shield, accommodates shoulders
Imaging free bore diameter	25 cm	ID of volume receive coils, accommodates heads
Gradient strength	0.3 × 0.3 × 0.6 T/m (x,y,z)	
FOV	24 × 24 × 30 cm (x,y,z)	FOVxy is covered using electromagnet shift fields, FOVz is covered using mechanical translation of the bed
Drive frequency	45.01 kHz	
Drive amplitude	≤ 3.5 mT peak	
Detection limit, volume coils	10 μg Fe (ferucarbotran) 130 μg Fe (ferumoxytol)	Sensitivity is approximately constant within the FOV
Detection limit, surface coils	500 ng Fe @ 1.5 cm distance (ferucarbotran)	Sensitivity changes with distance from the coil
Resolution, ferucarbotran	1–1.25 cm in x/y (20% MTF) est. 0.3 cm in z (20% MTF)	z resolution is estimated as the phantom did not test below 0.75 cm in z (90% measured MTF)
Resolution, ferumoxytol	est. >2 cm in x/y 1.25 cm in Z (20% MTF)	x,y resolution visually estimated as the phantom did not test above 2 cm
Dynamic range / shine-through	Demonstrated 8000:1 with surface coils	When using surface coils, coil positioning drives shine-through performance

**Extended Data Table 2:** Human study results & data model best-fit estimates.

(a) All human subjects, with & without magnetic tracer injection										
Body Part	Foot						Head			
Subject ID	S01	S02	S03	S06	S04	S05	S01	S06	S05	S04
**Age**	49	45	39	29	44	29	50	29	29	44
**Sex**	F	M	M	F	M	M	F	F	M	M
**Injected iron dose, volume**	n/a	n/a	n/a	n/a	**7.5 mg, 0.5 mL**	**15 mg, 1 mL**	n/a	n/a	**7.5 mg, 0.5 mL**	**15 mg, 1 mL**
**Imaging sessions**	1	1	1	1	6	6	1	1	7	6
**Study duration**	1 day	1 day	1 day	1 day	27 weeks	28 weeks	1 day	1 day	20 weeks	12 weeks
**PNS threshold, peak drive field**	>3.5 mT	>3.5 mT	>3.5 mT	>3.5 mT	>3.5 mT	>3.5 mT	>3.5 mT	>3.5 mT	>3.5 mT	3.2* mT
**Adverse events (CTCAE v5.0)**	none	none	none	none	skin discoloration (grade 1)	skin discoloration (grade 1)	none	none	skin discoloration (grade 1)	skin discoloration (grade 1), pain at injection site (grade 1)
(b) Human and animal pharmacokinetic best-fit parameter estimates								
		Human Foot		Human Head		Murine Foot		
		7.5 mg ferumoxytol (AMAG)	15 mg ferumoxytol (AMAG)	7.5 mg ferumoxytol (AMAG)	15 mg ferumoxytol (Sandoz)	n=7 ferumoxytol (AMAG)	n=3 ferumoxytol (Sandoz)	n=3 ferucarbotran (Meito)
**Injection site** $y=A_{1}e^{-t/\tau_{1}}+A_{2}e^{-t/\tau_{2}}$	$A_{1}$	4.9 mg	15.1 mg	2.8 mg	11.4 mg	78.3 %ID	81.0 %ID	27.2 %ID
	$\tau_{1}$	19.7 hr	11.1 hr	29.5 hr	18.7 hr	1.9 hr	1.8 hr	23.1 hr
	$A_{2}$	1.2 mg	0.8 mg	1.9 mg	4.4 mg	24.5 %ID	20.5 %ID	72.9 %ID
	$\tau_{\text{2}}$	23.7 weeks	25.3 weeks	18.6 weeks	20.1 weeks	8.3 weeks	6.9 weeks	10.3 weeks
**Lymphatics** $y=\frac{A\left(e^{-t/\tau_{1}}-e^{-t/\tau_{2}}\right)}{\left(1/\tau_{1}\right)-\left(1/\tau_{2}\right)}+C$	A	*N/A*	*N/A*	−0.0054 a.u./hr	−0.0082 a.u./hr	−79.8 %ID/day	−50.9 %ID/day	−75.3 %ID/day
	$\tau_{1}$	*N/A*	*N/A*	13.7 hr	14.1 hr	1.1 hr	2.2 hr	1.6 hr
	$\tau_{2}$	*N/A*	*N/A*	5.1 days	9.4 days	53.6 days	43.1 days	52.1 days
	C	*N/A*	*N/A*	0.021 a.u.	0.037 a.u.	Model set *C* = 0	Model set *C* = 0	Model set *C* = 0

* 50% probability from logistic curve fit

## Supplementary Material

Supplement 1

## Data Availability

Participant demographics, imaging parameters, and longitudinal model results are provided in the Article and Supplementary Information. Two anonymized MPI imaging datasets corresponding to Fig. 1 are provided in 3D Slicer-compatible format as Supplementary Data.

## Code Availability

As detailed in Methods, image quantitation and analyses were performed using standard methodologies; no code implementing novel analytical algorithms central to the main findings was developed. Image reconstruction used a previously published algorithm, described and cited in Methods.
