# Supplementary material for: Magnetic Particle Imaging in Human Subjects
Source: Res Sq. 2026 Mar 17:rs.3.rs-8825005. Preprint. [Version 1] doi: 10.21203/rs.3.rs-8825005/v1 (PMC13015610; doi:10.21203/rs.3.rs-8825005/v1)
Supplement: Supplement 1 [file NIHPPrs8825005v1-supplement-1.pdf]

Supplementary Methods

Black text is repeated from Methods. Blue text provides additional details only present in Supplementary Methods.

MPI Clinical Imager. An MPI image is acquired by moving a field-free region throughout a field of view, applying a drive field, and measuring signals induced in receive coils by magnetic tracers. The saturation of the tracer's magnetic response in a strong field enables spatial encoding of signals near the instantaneous position of the field free region[25,56,57] (**Extended Data Figure 1**). In this work, a field free point (FFP) is generated by a main magnet and shifted both magnetically and mechanically to cover the field of view. A drive assembly produces a pure sinusoidal field only within the field of view, and volume and surface receive coils detect the signal produced by the tracers. These subsystems are controlled using a realtime controller (National Instruments, Austin, TX) and all imaging is performed in a magnetically shielded room (ETS-Lindgren, Cedar Park, TX). Complete system specifications are provided in **Extended Data Table 1**.

The main magnet produces a strong gradient field containing a single point where the total magnetic field passes through zero, the FFP, and electronically shifts the FFP across a FOV (**Extended Data Figure 2,3**). The main magnet is constructed using four electromagnets arranged in a quadrupolar configuration on an iron yoke. The gradient is generated by applying dc currents, and transverse (x, y) shifting is performed by superimposing additional time-varying currents. The gradient strength at the FFP is 0.3 × 0.3 × 0.6 T/m, and typical shifting fields of +/- 36 mT allow sampling across an axial (x/y) slab up to 24 cm × 24 cm. The magnet is constructed using water-cooled hollow conductors, driven using current-controlled amplifiers (IECO Oy, Helsinki, Finland), and dissipates ~80 kW during operation. The FOV is sampled axially (z) in discrete slabs by mechanically stepping the subject bed position across a 30 cm range.

The drive field interacts with the magnetic tracer to produce a signal. The field is a homogenous, axially oriented (z), sinusoidally varying magnetic field at a frequency of 45.03 kHz and an amplitude of up to 3.5 mT peak at the isocenter (**Extended Data Figure 2,5**). The drive field (referred to interchangeably as "transmit") is produced by a water-cooled resonant solenoid coil and capacitor bank (Celem Capacitors, Jerusalem, Israel) mounted to the subject bed. The coil is powered by an industrial audio-frequency amplifier (8504, AE Techron, Elkhart, IN) whose output is filtered with a balanced, fourth order passive filter. The drive field signal chain dissipates up to 875 W, with 550 W dissipated into the transmit coil, 50 W dissipated in the bore due to induced eddy currents, and 275 W into the filter. During operation, active feedback control maintains transmit phase and amplitude.

Volume and surface receive coils inductively detect signals from magnetic tracers within the FOV. A pair of volume coils (**Extended Data Figure 5**), whose winding patterns are inspired by Hadamard encoding, are mounted concentrically inside the drive coil. The first receive coil is a 2-part (+1, -1) gradiometer, and the second receive coil is a 3-part gradiometer (+1/2, -1, +1/2), each wound to minimize feedthrough from the transmitter. Each gradiometer part has a typical operating voltage of up to ~1.4 kVp, and the sum of the parts cancels the voltage at the coil output to a manageable <10 Vp. While each volume coil individually has null regions within the field of view, the coils are designed with complementary sensitivity patterns so that when combined during reconstruction, they provide uniform signal coverage across the field of view without sensitivity null regions. In contrast to the uniform sensitivity of the volume coils, the sensitivity of the surface coils varies spatially and drops off with distance from the coil's surface, which can be used to selectively detect signals from a desired region of interest. Surface coils (**Extended Data Figure 6**) are mounted to a rigid application-specific head cradle positioned inside the volume coils. The cradle accurately locates up to four surface coils next to the cervical and auricular lymph node regions. Each surface

coil conductor is constructed as a printed circuit board and designed with a conductor winding optimized for high sensitivity at distances of 1.5-3 cm from the coil surface while minimizing coupling to the volume transmit coil. Human data presented utilized a left-side only coil configuration, as magnetic tracer injections were in the left parietal scalp and subjects showed unilateral lymphatic drainage. The voltages from the 4 receive channels, 2 volume and 2 surface, are filtered with a balanced-input passive notch filter (133 dB attenuation) to remove remaining feedthrough from the transmitter prior to amplification with a custom low-noise preamplifier with a differential input based on the JFE2140 (Texas Instruments, Dallas, Texas, USA).

An imaging pulse sequence acquires a 3D volume up to 24 × 24 × 30 cm by acquiring axial slabs with mechanical bed shifts between slabs (**Extended Data Figure 2c**). For each slab, a raster acquisition sequence applies a magnetic field gradient, shifts the FFP position electronically across x and y using a triangular waveform, and applies a drive field along z. A slab acquisition covers FOVxy = 20-24 cm, typically with y-direction fast shift at 4 traversals per second and an electronic raster step size of dx = 5 mm. Axial coverage (FOVz) is typically 10-28 cm with mechanical step size of dz = 5 mm to provide sufficient spatial encoding[58]. For FOVz = 28 cm, total scan time is approximately 15 minutes. Sequence parameters for all MPI images taken on the clinical scanner are reported in **Supplementary Table ST1**.

Magnetic Tracers. Phantom, preclinical, and clinical studies utilized ferumoxytol and the active ingredient in ferucarbotran, dextran magnetite (Meito Co. Ltd., Nagoya, Japan), which we refer to as ferucarbotran in this document. Ferumoxytol is an iron oxide coated with polyglucose sorbitol carboxymethylether with a 17-31 nm hydrodynamic diameter[29], and is approved for iron replacement therapy[29] and cancer neuroimaging[30]. Ferumoxytol was obtained from two manufacturers, AMAG Pharmaceuticals (Feraheme; Waltham, MA, USA) and Sandoz (generic; Basel, Switzerland). Ferucarbotran is an iron oxide coated with carboxydextran with a ~57 nm hydrodynamic diameter[47], and is approved for human use as an MRI contrast agent in Germany and Sweden (Resotran; B.E. Imaging GmbH, Baden-Baden, Germany)[59] and Japan (Resovist; PDR Pharma Co. Ltd., Tokyo, Japan)[60], and was not available for our study. Magtrace, an FDA-approved medical device for SLNB procedures[35] with MPI imaging performance comparable to ferucarbotran (data not shown), was also not available for our study.

Signal Processing & Image Reconstruction. The analog signals are digitized at 4 MSPS by the realtime controller and digitally filtered to isolate the 90-250 kHz frequency band. The time domain signals are transformed into harmonic portraits $d_k(x,y,z)$ (where k=harmonic number), by filtering harmonic bands of the drive field frequency and gridding them to the instantaneous FFP position (**Extended Data Figure 1b**). The harmonic portraits are reconstructed into volume or surface coil specific images using multi-harmonic gridded 3D deconvolution (MH3D)[58], which is a convolutional forward model that incorporates the transmit and receive coil sensitivity patterns and the magnetic tracer's point spread function.

The MH3D convolutional forward model[58] shapes the reconstruction point spread function and background noise such that integrated signal remains constant regardless of ROI size provided the signal is fully encompassed. This property enables quantitation using simple geometric ROIs rather than anatomically shaped regions.

For each scan, volume coil images and, when applicable, surface coil images are reconstructed. When both are used, the images are typically combined using coil sensitivity-weighting into a single composite image displayed in a perceptually linear false color colormap designed for MPI ("bluesteel", black-blue-gold, **Supplemental Figure S2, Supplemental Table ST2**). However, in the case of ferumoxytol at high dynamic range, low native resolution and low sensitivity worsen shine-through artifact and reconstruction

noise, making a single composite visualization challenging. Therefore, to better visualize the surface coil signal with this tracer, we display the volume coil data in grayscale and the surface coil data in bluesteel as separate overlays rather than compositing them into a single image. In all cases, resulting MPI images are displayed either as maximum intensity projections (MIPs) created using MATLAB (2024b, MathWorks, Natick MA) or as volume renderings co-registered with an optical scan or X-ray CT using 3D Slicer[61].

Basic Safety. The imager was verified using a risk-based approach against a subset of requirements defined by the IEC and FDA for MRI, in this work defined as "basic safety" testing. Our verifications included general requirements for medical equipment[62], with a focus on electrical safety. For MPI-specific verifications arising from the use of time-varying magnetic fields[63], we evaluated the imager against MRI guidance for Specific Absorption Rate (SAR) in order to support Nonsignificant Risk determination and performed magnetostimulation studies to determine stimulation thresholds in human heads and feet[64–66]. We detail the SAR and magnetostimulation testing below.

Specific Absorption Rate (SAR) Simulation and Phantom Testing. SAR was simulated on an anatomically realistic virtual human body model ("Duke") and a saline phantom using Sim4Life (ZMT Zurich MedTech AG) and experimentally verified for the phantom (**Figure 3**). See **Supplementary Methods** for details.

The simulations modeled only the drive field conductor, and simulated a 7 mT peak drive field, which was twice the field strength used in the study. Simulations were performed by Tesla Dynamic Coils (Zaltbommel, Netherlands).

SAR testing was performed by adapting standardized methods used in MRI (NEMA MS 8-2016[66]). The phantom was constructed using an LDPE carboy and insulated with Styrofoam. Due to the expected low SAR, the conductivity of the phantom was higher than physiological conductivity to allow for sufficient heating for reliable measurements. Measurements were performed on the insulated phantom at a 3.5 mT peak drive field utilizing a high accuracy thermometer and probe (TTI-10 Precision Thermometer, IsoTech, Colchester, Vermont), and the results compared with simulation.

Human PNS Threshold Simulation. Peripheral nerve stimulation (PNS) is the activation of nerves in response to a time-varying magnetic field. Electromagnetic simulations were performed to determine the onset of PNS in a virtual body model for the drive field conductor up to 7 mT peak. The simulation used the Sim4Life software and estimated the onset of PNS using the drive field conductor model used in the SAR simulations, the "Yoon-sun" virtual human body models, and the NEURON Solver implementing the "McIntyre-Richardson-Grill" (MRG) neuronal model.

Human Magnetostimulation Threshold Testing. The magnetostimulation threshold studies were designed to measure the onset of PNS and/or magnetophosphenes, primarily informing the selection of imaging sequence parameters. When experienced by a subject, the PNS stimulation at threshold is experienced as a perceptible activation of peripheral nerves such as light tingling or twitching, and magnetophenes at threshold are experienced as faint flickering visual sensations[64]. Prior experimental MPI human PNS testing focused on the drive fields[67–69]. Here we test all magnetic fields used in an imaging sequence. The study was approved by the Advarra (Protocol #Pro00071449) Institutional Review Board and the Stanford Institutional Review Board via reliance.

Exclusion criteria for the study was:

1. Active implants in any part of the body, including but not limited to: Pacemakers, drug ports with metal, implanted stimulator devices, including cochlear implants, deep brain stimulators, vagus nerve stimulators, other implanted electrodes or stimulators.
2. Indwelling medical devices that contain metal in the head, including but not limited to: Aneurysm clips or coils and stents.
3. Copper IUD.
4. Known or suspected pregnancy – a pregnancy test will be required for women of child-bearing potential.
5. History of work in the metal working industry.
6. History of known or suspected orbital metal foreign bodies.
7. History of claustrophobia.
8. History of migraines, epilepsy, or seizure.
9. History of heart rhythm disturbances.
10. Weight greater than 250 lbs.
11. Unable to comfortably fit inside the imager region where the magnetic fields are generated and detected.

Additional exclusion criteria for foot testing:

1. Unconfirmed MRI-compatible indwelling medical devices containing metal and non-removable metal objects on the foot or leg that will be scanned, including but not limited to:
   (a) Pins, plates, screws, etc.
   (b) Non-removable piercings and metal fragments.
2. Tattoos on the foot or leg that will be scanned.

Additional exclusion criteria for the head testing:

1. Unconfirmed MRI-compatible oral devices; stainless steel crowns, orthodontic devices such as permanent retainers, braces and wires, and dental implants.
2. Unconfirmed MRI-compatible indwelling medical devices containing metal and non-removable metal objects, in the head and neck, including but not limited to:
   (a) Pins, plates, screws, etc.
   (b) Non-removable piercings, metal fragments, and certain kinds of hair extensions.
3. Tattoos on the neck and head, including permanent makeup.

MRI compatibility was determined by a subject history of MRI imaging since the object was placed or by referencing the MRI compatibility database on MRIsafety.com (e.g., for example amalgam fillings are considered "safe" and were allowed for head visits).

Magnetostimulation thresholds were measured in 6 healthy adult subjects (n=6 feet, n=4 head). The study used a systematic protocol to isolate effects of each magnetic field component for the gradient, shift, drive field, as well as combinations of all the fields. For each configuration, field amplitude was first increased in coarse steps up to the maximum amplitude. During testing, subjects reported any sensations or visual perceptions. In the event of stimulation, the process is then repeated with finer step sizes starting below the coarse threshold. Last, the subject is tested with random amplitudes within ±15% of the fine threshold. Because higher frequencies (e.g., >10 kHz) tend not to have a sharp transition at the stimulation threshold, this fine sampling and statistical modeling approach enables accurate and consistent measurement of the amplitude at which a subject has a 50% probability of stimulation[67]. To determine this 50% probability amplitude, the binary magnetostimulation data was fit to a logistic regression (**Figure 3f**).

Subjects notified study personnel of magnetostimulation via a Bluetooth-enabled button, verbally confirmed stimulation had occurred, and described the location and description of sensation to the study per-

sonnel. During each session, subjects were monitored by the study doctor and a study coordinator who maintained two-way audio communication with the system operator. During scans, subjects were provided with a non-magnetic, MPI-compatible, emergency alert squeeze bulb (MagnaAlarm Panic Alarm, Magnacoustics).

Study demographics and results are shown in **Extended Data Table 2** and **Figure 3**. For subjects that experienced magnetostimulation, results were used to set field amplitudes for subsequent imaging experiments.

Human Imaging with Tracer Injection. A subset of subjects enrolled in optional human imaging with tracer injection substudies. The substudy enrolled 2 healthy adult subjects (n=2 feet, n=2 head). The study was approved by the Advarra (Protocol #Pro00071449) Institutional Review Board and the Stanford Institutional Review Board via reliance. Adverse events were classified using the Common Terminology Criteria for Adverse Events (CTCAE) v5.0[70]; if the adverse event was not specifically listed in the CTCAE, the general grading guidelines were applied.

Additional exclusion criteria for the tracer injection study beyond those seen in the magnetostimulation study included:

1. History of iron overload disease.
2. Known intolerance/hypersensitivity to iron, and/or dextran compounds, and/or Magtrace.
3. Known hypersensitivity to ferumoxytol or any of its components.
4. History of diagnosed impaired lymphatic function.
5. History of iron replacement products infusion (e.g., ferumoxytol) injection within the past 12 months.
6. Participation in an investigational drug study within 30 days from date of consent.
7. Currently breastfeeding/lactating women.

For the head injections (n=2), subcutaneous injections were performed in the parietal scalp. For the foot injections (n=2), subcutaneous injections were performed in the lateral heel, between the lateral malleolus and Achilles tendon. The tracer injected in all cases was ferumoxytol, manufactured by either AMAG Pharmaceuticals or Sandoz (see table below). All dilutions were performed immediately prior to injection in a 1:1 ratio with sterile saline. All injections were performed by a physician at Stanford University Medical School, Stanford, California, USA.

Injection details by anatomy and subject:

Anatomy	Subject	Ferumoxytol Source	Iron Quantity	Injection Volume
Head	S05	AMAG Pharma.	7.5 mg	0.5 mL
Head	S04	Sandoz	15.0 mg	1.0 mL
Foot	S04	AMAG Pharma.	7.5 mg	0.5 mL
Foot	S05	AMAG Pharma.	15.0 mg	1.0 mL

Following injection, the subject was monitored for 30 minutes, then transported to the study site and first underwent repeat magnetostimulation protocol, resulting in a ~2 hr delay before the first imaging timepoint.

For subjects that experienced magnetostimulation, imaging was typically performed at one to two standard deviations below the 50% probability amplitude, and no imaging was performed beyond 50%. Specifically, for the subject (S04) that experienced magnetostimulation in the head, most images were acquired at a 2.8 or 2.4 mT peak (50%-1σ or -2σ) drive field amplitidue, and no imaging was performed beyond $<$3.2 mT peak (50%). All other subjects (head and feet) did not experience magnetostimulation and used the maximum drive amplitude of 3.5 mT peak.

Longitudinal imaging was performed via multiple imaging sessions post-injection. To capture expected tracer clearance rates, imaging sessions occurred more frequently in the week following injection (day 1–3, etc.) and then more widely spaced through the experimental window (1 week–6 months). While consistent sequence parameters were targeted for longitudinal imaging of injection sites, the exploratory nature of the lymphatic imaging resulted in some variation in sequence parameters. Sequence parameters for all images used in the analysis are specified in **Supplementary Table ST1**.

The injection site was photographed after each imaging session to monitor skin discoloration. Subjects were scanned with a 3D camera (Einstar Vega, Shining 3D, Hangzhou, China). MPI and optical scans were co-registered using MPI/optically-visible fiducials in 3D Slicer[61].

Human Data Image Analysis & Quantitative Calibration. Injection sites and lymphatic regions were analyzed using region-specific image processing workflows. For the injection site, signal was quantified over time and calibrated to quantify magnetic tracer quantity over time. For the lymphatic region, signal was quantified over time but not calibrated, due to higher signal variability caused by scan-to-scan changes in subject positioning and in imaging sequence parameters, complicated by the field nonuniformity in the surface coils.

Injection site signal quantification was performed by integrating the signal over a 3D rectangular prism ROI_{Inj} (10 × 10 × 4 cm) of the volume coil reconstruction (V) for each image in the time series (**Figure 2a**).

Injection site signal calibration established an integrated signal-to-tracer quantity scaling factor using a phantom modeling the injection site. The phantom was constructed by loading a 2.25 cm disc-shaped cotton absorbent pad with tracer matching the subject's injection quantity and volume. The pad was used to mimic the distribution of tracer following subcutaneous injections. It was placed in a resealable plastic sleeve, and affixed to a head phantom at the approximate injection site location for imaging using the same sequence and parameters.

Additional approaches for injection site signal calibration were tested before selecting the pad-phantom scaling factor. These approaches included placing a known quantity of tracer within the field of view, and measuring a signal-to-tracer scaling curve using a dilution series. For a known quantity calibration, reference fiducials were attached to the subject during imaging (e.g., "Fid" in **Figure 1f**); however, the large signal from the higher-dose injection sites sometimes washed out signal from the fiducial, reducing calibration accuracy. For measuring a signal-to-tracer scaling curve, a serial dilution of reference vials was imaged that spanned the expected iron quantities in the image (0.625–20 mg Fe). While the dilution series confirmed linear system response, we found that the current reconstruction algorithms produce systematic quantitation errors when applied to different geometries such as point sources and broad discs.

Head-and-neck lymph node region signal quantification was performed by integrating the signal over a 3D rectangular prism ROI_{LNs} of the surface coil reconstruction (S) for each image in the time series **(Figure 2a)**. Only the surface coil reconstruction was used, as the volume coils lacked sufficient sensitivity and shine-through performance for imaging ferumoxytol in the lymph nodes. With ferumoxytol's low spatial resolution, individual lymph nodes were not expected to be resolvable, so signal was integrated over the entire region rather than reported for individual nodes. The ROI_{LNs} dimensions (S04: 14 × 14 × 12 cm, S05: 12 × 14 × 10 cm) were set for each subject, and consistent across all longitudinal time points (**Supplemental Table ST1)**.

The team experimented with adjusting sequence parameters and subject positions to identify and confirm lymphatic signal. To achieve a grouping of datasets with consistent imaging parameters for longitudinal

analysis, images were selected with defined ranges of FOVz, z step size, application-specific surface coil position, and head rotations. The remaining imaging parameter variation within these ranges likely contributes to the variance observed in the lymphatic signal time points.

Time-Series Data Analysis. Time-series analysis was performed by fitting parametric models in MATLAB (2024b, MathWorks, Natick MA). The injection site was modeled assuming monotonic tracer clearance. Lymphatic uptake and clearance were modeled assuming signal accumulation and decay occur concurrently. Best-fit parameter estimates are reported in **Extended Data Table 2b**. The time-series analyses were performed independently for the injection site and lymph node regions. Sequence parameters for all images used in each analysis are listed by figure in **Supplemental Data Table ST1**.

Injection site clearance dynamics were modeled by a bi-exponential decay, and the decay time values are reported for a least-squares fit to measured data using a solver built on nonlinear Nelder-Mead optimization (*fminsearch*). Prediction intervals (95%) were estimated using a parametric bootstrap with Student-t noise and a small-sample variance correction to avoid undercoverage given limited data points[71]. The images used in the injection site time-series analysis were acquired using a predefined imaging sequence and subject position.

Bi-exponential decays were modeled as:

$$y = A_1 e^{-t/\tau_1} + A_2 e^{-t/\tau_2} \tag{1}$$

where A_1 and A_2 are amplitudes and τ_1 and τ_2 are the fast and slow time constants.

Lymphatic update and clearance dynamics were modeled by a two-pole overdamped model, which can be shown equivalent to a two-compartment model[72], and the time constants are reported for a least-squares fit to measured data. Parameters and their 95% prediction intervals were estimated by nonlinear least-squares regression (*fitnlm*). The images used in the lymphatic time-series analysis pooled a few groupings (detailed in **Supplementary Table ST1**) of imaging sequence parameters and head rotation positions (positions illustrated in **Extended Data Figure 7a**). The pooling balanced time-course sampling density with the high image-to-image parameter variability. Error bars in **Figure 2d** show the average and standard deviation for images grouped by same acquisition day.

The two-pole overdamped system is modeled as:

$$y = \frac{A\left(e^{-t/\tau_1} - e^{-t/\tau_2}\right)}{(1/\tau_1) - (1/\tau_2)} + C, \tag{2}$$

where $-1/\tau_1$ and $-1/\tau_2$ are distinct real poles, A is the response amplitude and C is an optional constant offset.

Imaging Performance Characterization. The system performance was characterized for spatial resolution, shine-through, and sensitivity using phantoms with both ferumoxytol and ferucarbotran (see **Extended Data Figures 4-6** and **Supplementary Methods** for details).

Spatial resolution was characterized with a Derenzo phantom containing wells of diameter 0.75–2.00 cm, with 0.70 cm depth (**Extended Data Figure 4**). In order to test image resolution at tracer concentrations

that balance tracer signal and system noise, tracers were diluted with saline such that the smallest well (d = 0.75 cm) contained ~10× above the detection limit. Accordingly, ferucarbotran was diluted to 0.3 mg/mL (93 µg/well), and ferumoxytol to 1.6 mg/mL (496 µg/well). Images were acquired for three orientations to capture anisotropic resolution: axial for x/y, two coronal rotations for z and an additional x measurement. Modulation transfer function (MTF) values were then calculated from line profiles by measuring signal at wells and midpoints between wells.

Shine-through performance, which is the ability to resolve a small signal nearby a larger signal source, is tested in phantoms using two configurations. This contrasts with imager dynamic range performance, which is the ratio of the largest to smallest signals that can be imaged in isolation. The first configuration tested the lowest resolution dimensions (x/y) with only the volume coils. A phantom was constructed consisting of an axial spiral arrangement of samples (140 µg ferucarbotran) around a 20× larger tracer source (2.8 mg), at distances of 1.7-6.1 cm, similar to phantom testing performed for Sentimag wand with Magtrace[73]. An image of the phantom was acquired and assessed by inspecting line profiles (**Extended Data Figure 5e**). The second configuration tested anatomically inspired geometries using the lymph node head-and-neck phantom used for benchmarking MPI performance against nuclear medicine (see below). Images were acquired for phantoms containing tracer ratios of 100:1 (**Figure 4**, ferucarbotran and ferumoxytol) and 8000:1 (**Extended Data Figure 6d**, ferucarbotran).

Volume coil sensitivity and linearity vs concentration were assessed using serial 2× dilutions ([72, 36, 18, 9.0, 4.5, 2.3] µg ferucarbotran; [800, 400, 200,100, 50, 25] µg ferumoxytol) for 0.1 mL samples at three positions (n=3) distributed within the field of view to capture any spatial shift variance (**Extended Data Figure 5f,g**). Our reconstruction (MH3D) shapes the image noise statistics, resulting in non-Gaussian and spatially correlated noise. Thus, a suitable metric for detection threshold was defined as the point at which the maximum sample signal, averaged across the three samples (peak signal), matched the image background artifact level (peak background). Peak signal was measured from 3D ROIs centered at each sample, yielding three values per image that were then averaged. The peak background ROI was defined as all values outside the peak signal ROIs. Detection was calculated as the ratio of peak signal to peak background artifact, with detection defined as a ratio ≥ 1.

Application-specific surface coil sensitivity and linearity were assessed using serial 2× dilutions (18 µg–500 ng ferucarbotran) for four samples positioned 1.5 cm from each of four surface coils (**Extended Data Figure 6c**). The surface coils exhibit strong spatial sensitivity variation, which limited our ability to perform consistent detection threshold calculations using MH3D. We found that image processing at the intermediate stage of harmonic portraits ($2f_0$) yielded SNR measures that behaved as expected for Gaussian noise, enabling standard peak SNR (pSNR) measurements. Peak signal was measured from ROIs centered at each sample, yielding four measurements per concentration that were then averaged. pSNR was calculated as the ratio of average peak signal to background standard deviation, with detection defined at pSNR = 3.3 (99.9% confidence).

Imaging Performance Benchmarking with SPECT and Gamma Camera. Phantom studies were completed to benchmark MPI image quality against Tc-99m scintigraphy and SPECT in head and neck lymphatic imaging. A phantom was constructed from a Styrofoam mannequin head (SmoothFōM® Male Foam Head, FloraCraft®, Ludington, Michigan) with holes for a 1.5 mL tube in the left parietal scalp to model the injection site, and eleven 0.2 mL tubes in periauricular and cervical regions to model draining lymph nodes on the same (left). Images were acquired on the phantoms using planar scintigraphy, SPECT, MPI with ferucarbotran and MPI with ferumoxytol. See **Supplementary Methods** for details.

Two replicates of the phantom were built to facilitate imaging across collaborating institutions, but manual

production resulted in 2-4 mm inter-model difference for the lymph node locations in the phantoms used for MPI and for nuclear medicine. Both magnetic and radiotracer phantoms were filled with a 100:1 tracer quantity ratio between the injection site and lymph node vials for comparison.

To prepare the phantom for SPECT and planar scintigraphy, vials were loaded with [^{99m}Tc]tilmanocept (Lymphoseek; Cardinal Health, Dublin, OH, USA) radiotracer. The injection site vial was filled with the FDA-recommended dose[48] of 0.5 mCi in 0.5 mL. The 11× lymph node vials were each filled with 5 µCi, selected based on data from clinical studies that report approximately 0.5–1.8% of injected dose is accumulated[50]. Planar scintigraphy and SPECT imaging (Intevo Bold, SIEMENS) immediately followed phantom preparation. Planar scintigraphy used a 5-minute acquisition, 256 × 256 matrix, LEHR collimator, 140 keV with 15% window but no scatter window setting. SPECT imaging employed 64 stops, 22 sec per stop, non-circular orbit, step and shoot, 128×128 matrix, LEHR collimator, 140 keV with 15% window, and a 15% lower scatter window.

To prepare the phantom for MPI imaging, the vials were filled with ferucarbotran or ferumoxytol magnetic tracer. The injection site vial was filled with 14 mg tracer in 0.5 mL, similar to the injected doses of ferumoxytol used for humans in this study, and to doses used for studies with magnetic tracers Magtrace and Sienna+ for use in melanoma[74] and oral cancer[51]. The 11× lymph node vials were each filled with 140 µg of the corresponding magnetic tracer, based on reported lymph node accumulation[49,51]. In a second experiment, to test the limits of MPI imaging with application-specific receiver coils, ferucarbotran was loaded at a higher ratio of 8000:1, with 56 mg in the injection site vial and 7 µg in each lymph node vial (**Extended Data Figure 6d**). MPI image acquisitions used similar sequences to those used for human imaging and are detailed in **Supplementary Table ST1**.

Preclinical Animal Testing & Tracer Relaxometry. Mouse tracer pharmacokinetics studies were conducted at the Robarts Research Institute under an approved protocol from Western University's Institutional Animal Care and Use Committee (Protocol #2023-113) and adhered to ARRIVE guidelines. Healthy female C57BL/6 mice (12–14 weeks of age, 20–25 g; Charles River) were obtained and cared for in accordance with the standards of the Canadian Council on Animal Care.

Mice received subcutaneous hind-footpad injections of one of three tracers: ferumoxytol manufactured by AMAG Pharmaceuticals (50 µg, n=7), ferumoxytol manufactured by Sandoz (50 µg, n=3), or ferucarbotran (25 µg, n=6) in a volume of 25 µL. Tracer administration was performed using a 30-gauge needle.

MPI images were acquired on a preclinical MPI scanner (Momentum, Magnetic Insight Inc., Alameda, CA) at multiple time points up to 8 weeks post-injection to quantify magnetic tracer pharmacokinetics. For *in vivo* MPI, the imaging field of view was 12 × 6 cm, with multichannel transmit along the x and z axes. The gradient strength was 5.7 T/m, and drive field amplitudes were 20 mT peak (x) and 23 mT peak (z), with a projection scan time of 2 minutes. Images were reconstructed using X-space methods[25,56,57].

MPI signal at injection sites and primary draining lymph nodes were quantified using an ROI with a threshold of $\geq$0.5× maximum intensity. For each timepoint, draining popliteal lymph node and injection site signals were normalized to the baseline MPI signal at the injection site on the ipsilateral limb to compute a percent injected dose (%ID). For time-course analysis, injection site and lymph node time series were fit with the same parametric models as for the human data. Data from all mice within each magnetic tracer group were pooled, and plots report the mean across animals with standard deviation error bars.

At study endpoint, mice were euthanized and the popliteal and iliac lymph nodes were excised and processed for Perl's Prussian blue staining to confirm iron deposition. The excised lymph nodes were fixed in 4% paraformaldehyde, paraffin-embedded, and sectioned into 5 µm slices.

Magnetic properties of ferumoxytol (AMAG Pharmaceuticals vs. Sandoz) were characterized using magnetic particle relaxometry (RELAX module, Momentum™, Magnetic Insight Inc, Alameda, CA). For relaxometry, ferumoxytol (75 µg) was placed in the center of the imaging holder. This concentration was optimized to produce peak signal = 1 a.u. to provide adequate SNR without saturating the detector. Relaxometry scans were performed using a drive field 20 mT and bias field 160 mT. The point spread functions were normalized to iron concentration for each magnetic tracer.

Supplementary Figures S1-S2

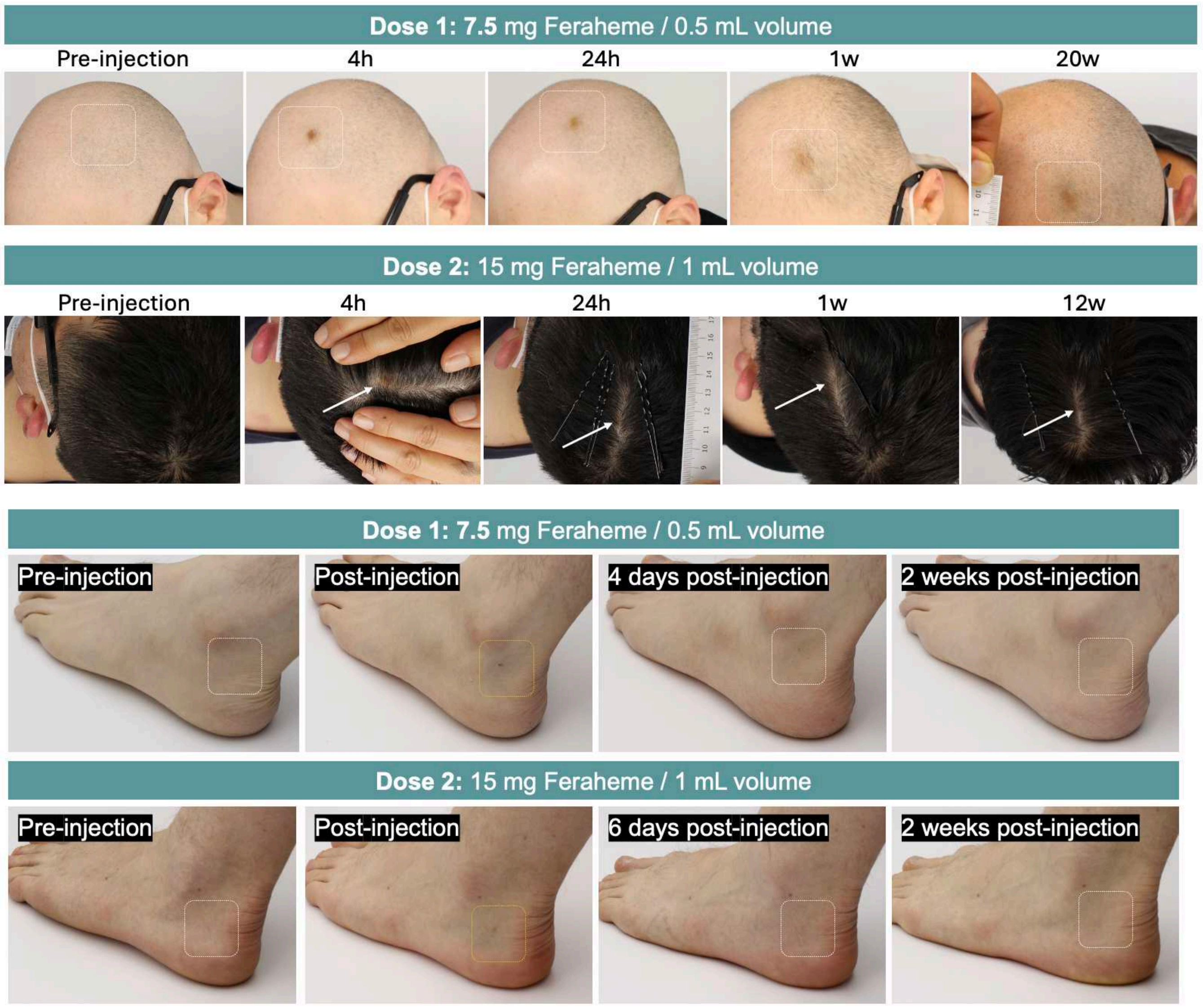

Supplementary Figure S1: Mild skin discoloration following subcutaneous magnetic tracer injection occurred at all injection sites (CTCAE grade 1, <10% body surface area).

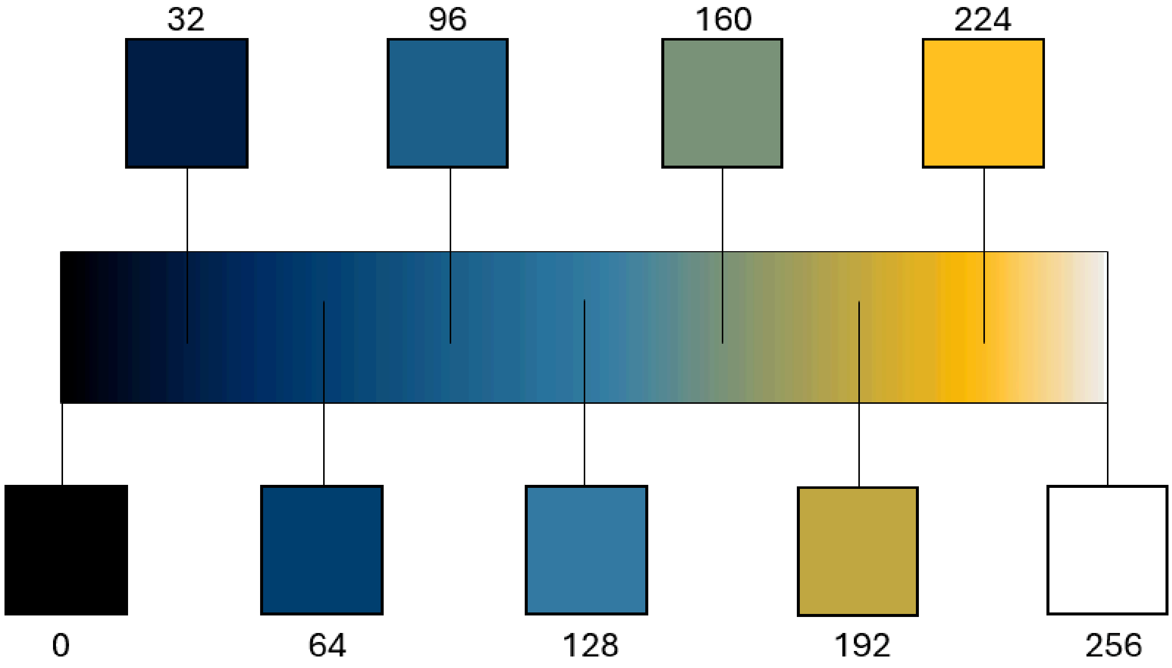

Supplementary Figure S2: Visual "bluesteel" color map.

Supplementary Tables ST1-ST2

Supplementary Table ST1: Clinical scanner image scanning parameters and internal identifier (UID).

Unique descriptor	Figure	UID	time after injection [hr]	time after injection [week]	FOVx [m]	FOVy [m]	FOVz [m]	fast shift axis	Ny or Nx	raster step size (dx or dy) [m]	Nz	dz [m]	shift traversals per sec	Drive amplitude [mTp]	app coil center z position [m]	app coil ch1	app coil ch2
S05 head (7.5 mg) coreg	1f	20250318-013429	296.775	1.767	0.22	0.22	0.26	y	46	0.005	53	0.005	4	3.5	-0.1	Left, Bottom	Left, Top
S04 head (15 mg) coreg	1g	20250430-203259	28.516	0.17	0.22	0.22	0.28	y	46	0.005	58	0.005	4	2.8	-0.11	Left, Bottom	Left, Top
S04 head (15 mg) coreg, w/ ROIs	2a	20250430-203259	28.516	0.17	0.22	0.22	0.28	y	46	0.005	58	0.005	4	2.8	-0.11	Left, Bottom	Left, Top
S05 foot (15 mg) coreg	2c (inset)	20250226-181520	1.856	0.011	0.22	0.22	0.2	y	46	0.005	41	0.005	4	3.5	0	N/A	N/A
S04 Foot 1	2c (7.5 mg)	20250113-184909	2.203	0.013	0.22	0.22	0.2	y	46	0.005	41	0.005	4	3.5	0	N/A	N/A
S04 Foot 2	2c (7.5 mg)	20250113-194046	3.063	0.018	0.22	0.22	0.2	y	46	0.005	41	0.005	4	3.5	0	N/A	N/A
S04 Foot 3	2c (7.5 mg)	20250113-203751	4.014	0.024	0.22	0.22	0.2	y	46	0.005	41	0.005	4	3.5	0	N/A	N/A
S04 Foot 4	2c (7.5 mg)	20250113-214609	5.153	0.031	0.22	0.22	0.2	y	46	0.005	41	0.005	4	3.5	0	N/A	N/A
S04 Foot 5	2c (7.5 mg)	20250113-224517	6.138	0.037	0.22	0.22	0.2	y	46	0.005	41	0.005	4	3.5	0	N/A	N/A
S04 Foot 6	2c (7.5 mg)	20250114-000858	7.533	0.045	0.22	0.22	0.2	y	46	0.005	41	0.005	4	3.5	0	N/A	N/A
S04 Foot 7	2c (7.5 mg)	20250114-013815	9.021	0.054	0.22	0.22	0.2	y	46	0.005	41	0.005	4	3.5	0	N/A	N/A
S04 Foot 8	2c (7.5 mg)	20250117-214438	101.127	0.602	0.22	0.22	0.2	y	46	0.005	41	0.005	4	3.5	0	N/A	N/A
S04 Foot 9	2c (7.5 mg)	20250127-173903	337.034	2.006	0.22	0.22	0.2	y	46	0.005	41	0.005	4	3.5	0	N/A	N/A
S04 Foot 10	2c (7.5 mg)	20250415-203451	2211.964	13.166	0.22	0.22	0.2	y	46	0.005	41	0.005	4	3.5	0	N/A	N/A
S04 Foot 11	2c (7.5 mg)	20250415-221540	2213.644	13.176	0.22	0.22	0.2	y	46	0.005	41	0.005	4	3.5	0	N/A	N/A
S04 Foot 12	2c (7.5 mg)	20250723-191419	4586.622	27.301	0.22	0.22	0.2	y	46	0.005	41	0.005	4	3.5	0	N/A	N/A
S04 Foot 13	2c (7.5 mg)	20250723-193104	4586.901	27.303	0.22	0.22	0.2	y	46	0.005	41	0.005	4	3.5	0	N/A	N/A
S04 Foot calibration pad phantom	2c (7.5 mg)	20250114-231246	N/A	N/A	0.22	0.22	0.2	y	46	0.005	41	0.005	4	3.5	0	N/A	N/A
S05 Foot 1	2c (15 mg)	20250226-183149	2.13	0.013	0.22	0.22	0.2	y	46	0.005	41	0.005	4	3.5	0	N/A	N/A
S05 Foot 2	2c (15 mg)	20250226-202916	4.088	0.024	0.22	0.22	0.2	y	46	0.005	41	0.005	4	3.5	0	N/A	N/A
S05 Foot 3	2c (15 mg)	20250227-175929	25.591	0.152	0.22	0.22	0.2	y	46	0.005	41	0.005	4	3.5	0	N/A	N/A
S05 Foot 4	2c (15 mg)	20250228-042743	36.062	0.215	0.22	0.22	0.2	y	46	0.005	41	0.005	4	3.5	0	N/A	N/A
S05 Foot 5	2c (15 mg)	20250228-161649	47.88	0.285	0.22	0.22	0.2	y	46	0.005	41	0.005	4	3.5	0	N/A	N/A
S05 Foot 6	2c (15 mg)	20250228-183231	50.142	0.298	0.22	0.22	0.2	y	46	0.005	41	0.005	4	3.5	0	N/A	N/A
S05 Foot 7	2c (15 mg)	20250304-224819	150.405	0.895	0.22	0.22	0.2	y	46	0.005	41	0.005	4	3.5	0	N/A	N/A
S05 Foot 8	2c (15 mg)	20250312-192334	338.993	2.018	0.22	0.22	0.2	y	46	0.005	41	0.005	4	3.5	0	N/A	N/A
S05 Foot 9	2c (15 mg)	20250722-181050	3505.781	20.868	0.22	0.22	0.2	y	46	0.005	41	0.005	4	3.5	0	N/A	N/A
S05 Foot 10	2c (15 mg)	20250722-184614	3506.371	20.871	0.22	0.22	0.2	y	46	0.005	41	0.005	4	3.5	0	N/A	N/A
S05 Foot 11	2c (15 mg)	20250911-211143	4732.795	28.171	0.22	0.22	0.2	y	46	0.005	41	0.005	4	3.5	0	N/A	N/A
S05 Foot 12	2c (15 mg)	20250911-215405	4733.501	28.176	0.22	0.22	0.2	y	46	0.005	41	0.005	4	3.5	0	N/A	N/A
S05 Foot 13	2c (15 mg)	20250911-220948	4733.763	28.177	0.22	0.22	0.2	y	46	0.005	41	0.005	4	3.5	0	N/A	N/A
S05 Foot calibration pad phantom	2c (15 mg)	20250304-023403	N/A	N/A	0.22	0.22	0.2	y	46	0.005	41	0.005	4	3.5	0	N/A	N/A
S05 Head 1	2b (7.5 mg)	20250305-185453	2.115	0.013	0.22	0.22	0.28	y	46	0.005	58	0.005	4	3.5	0	N/A	N/A
S05 Head 2	2b (7.5 mg)	20250305-210806	4.335	0.026	0.22	0.22	0.28	y	46	0.005	58	0.005	4	3.5	-0.08	Left, Bottom	Left, Top
S05 Head 3	2b (7.5 mg)	20250305-230551	6.298	0.037	0.22	0.22	0.28	y	46	0.005	58	0.005	4	3.5	-0.09	Left, Bottom	Left, Top
S05 Head 4	2b (7.5 mg)	20250306-011612	8.47	0.05	0.22	0.22	0.28	y	46	0.005	58	0.005	4	3.5	-0.08	Left, Bottom	Left, Top
S05 Head 5	2b (7.5 mg)	20250306-180010	25.203	0.15	0.22	0.22	0.28	y	46	0.005	58	0.005	4	3.5	-0.09	Left, Bottom	Left, Top
S05 Head 6	2b (7.5 mg)	20250307-022419	33.605	0.2	0.22	0.22	0.28	y	46	0.005	58	0.005	4	3.5	-0.1	Left, Bottom	Left, Top
S05 Head 7	2b (7.5 mg)	20250307-165235	48.076	0.286	0.22	0.22	0.28	y	46	0.005	58	0.005	4	3.5	-0.1	Left, Bottom	Left, Top
S05 Head 8	2b (7.5 mg)	20250307-191651	50.481	0.3	0.22	0.22	0.28	y	46	0.005	58	0.005	4	3.5	-0.09	Left, Bottom	Left, Top
S05 Head 9	2b (7.5 mg)	20250312-170959	168.366	1.002	0.22	0.22	0.28	y	46	0.005	58	0.005	4	3.5	-0.1	Bilateral, Bottom	Bilateral, Top
S05 Head 10	2b (7.5 mg)	20250317-215722	293.156	1.745	0.22	0.22	0.28	y	46	0.005	58	0.005	4	3.5	-0.1	Left, Bottom	Left, Top
S05 Head 11	2b (7.5 mg)	20250722-203935	3339.86	19.88	0.22	0.22	0.28	y	46	0.005	58	0.005	4	3.5	-0.1	Left, Bottom	Left, Top
S05 Head 12	2b (7.5 mg)	20250723-015010	3345.036	19.911	0.22	0.22	0.28	y	46	0.005	58	0.005	4	3.5	-0.08	Left, Bottom	Left, Top
S05 Head calibration pad phantom	2b (7.5 mg)	20250305-014042	N/A	N/A	0.22	0.22	0.28	y	46	0.005	58	0.005	4	3.5	-0.11	Left, Bottom	Left, Top
S04 Head 1	2b (15 mg)	20250429-192836	3.443	0.02	0.22	0.22	0.28	y	46	0.005	58	0.005	4	2.4	-0.11	Left, Bottom	Left, Top
S04 Head 2	2b (15 mg)	20250429-215150	5.831	0.035	0.22	0.22	0.28	y	46	0.005	58	0.005	4	2.4	-0.11	Left, Bottom	Left, Top
S04 Head 3	2b (15 mg)	20250430-163514	24.554	0.146	0.22	0.22	0.28	y	46	0.005	58	0.005	4	2.4	-0.11	Left, Bottom	Left, Top
S04 Head 4	2b (15 mg)	20250430-233117	31.488	0.187	0.22	0.22	0.28	y	46	0.005	58	0.005	4	2.4	-0.11	Left, Bottom	Left, Top
S04 Head 5	2b (15 mg)	20250501-164333	48.693	0.29	0.22	0.22	0.28	y	46	0.005	58	0.005	4	2.4	-0.11	Left, Bottom	Left, Top
S04 Head 6	2b (15 mg)	20250506-201245	172.179	1.025	0.22	0.22	0.28	y	46	0.005	58	0.005	4	2.4	-0.11	Left, Bottom	Left, Top
S04 Head 7	2b (15 mg)	20250506-204241	172.678	1.028	0.2375	0.22	0.28	y	50	0.005	58	0.005	4	2.4	-0.11	Left, Bottom	Left, Top
S04 Head 8	2b (15 mg)	20250723-215208	2045.836	12.178	0.2375	0.22	0.28	y	50	0.005	58	0.005	4	2.4	-0.11	Left, Bottom	Left, Top
S04 Head calibration pad phantom	2b (15 mg)	20250502-005105	56.818	0.338	0.22	0.22	0.28	y	46	0.005	58	0.005	4	2.4	-0.11	Bilateral, Bottom	Bilateral, Top
S05 Pos3 105/100/110 4-5mm 1	2d (7.5 mg)	20250307-022419	33.605	0.2	0.22	0.22	0.28	y	46	0.005	58	0.005	4	3.5	-0.1	Left, Bottom	Left, Top
S05 Pos3 105/100/110 4-5mm 2	2d (7.5 mg)	20250307-185400	50.1	0.298	0.22	0.22	0.1	y	46	0.005	26	0.004	4	3.5	-0.11	Left, Bottom	Left, Top
S05 Pos3 105/100/110 4-5mm 3	2d (7.5 mg)	20250312-223656	173.816	1.035	0.22	0.22	0.16	y	46	0.005	41	0.004	4	3.5	-0.105	Left, Bottom	Left, Top
S05 Pos3 105/100/110 4-5mm 4	2d (7.5 mg)	20250313-002923	175.69	1.046	0.22	0.22	0.1	y	46	0.005	26	0.004	4	3.5	-0.105	Left, Bottom	Left, Top
S05 Pos3 105/100/110 4-5mm 5	2d (7.5 mg)	20250317-224726	293.991	1.75	0.22	0.22	0.1	y	46	0.005	26	0.004	4	3.5	-0.1	Left, Bottom	Left, Top
S05 Pos3 105/100/110 4-5mm 6	2d (7.5 mg)	20250318-013429	296.775	1.767	0.22	0.22	0.26	y	46	0.005	53	0.005	4	3.5	-0.1	Left, Bottom	Left, Top
S05 Pos3 105/100/110 4-5mm 7	2d (7.5 mg)	20250722-205955	3340.199	19.882	0.22	0.22	0.26	y	46	0.005	53	0.005	4	3.5	-0.1	Left, Bottom	Left, Top
S05 Pos3 105/100/110 4-5mm 8	2d (7.5 mg)	20250722-211715	3340.488	19.884	0.22	0.22	0.1	y	46	0.005	26	0.004	4	3.5	-0.1	Left, Bottom	Left, Top
S05 Pos3 105/100/110 4-5mm 9	2d (7.5 mg)	20250722-235635	3343.143	19.9	0.22	0.22	0.26	y	46	0.005	53	0.005	4	3.5	-0.11	Left, Bottom	Left, Top
S05 Pos3 105/100/110 4-5mm 10	2d (7.5 mg)	20250724-002429	3367.608	20.045	0.22	0.22	0.26	y	46	0.005	53	0.005	4	3.5	-0.1	Left, Bottom	Left, Top
S05 PosN 105/100/110 4-5mm 1	2d (7.5 mg)	20250305-213342	4.762	0.028	0.22	0.22	0.28	y	46	0.005	58	0.005	4	3.5	-0.11	Left, Bottom	Left, Top
S05 PosN 105/100/110 4-5mm 2	2d (7.5 mg)	20250307-165235	48.076	0.286	0.22	0.22	0.28	y	46	0.005	58	0.005	4	3.5	-0.1	Left, Bottom	Left, Top
S05 PosN 105/100/110 4-5mm 3	2d (7.5 mg)	20250307-184534	49.959	0.297	0.22	0.22	0.1	y	46	0.005	26	0.004	4	3.5	-0.11	Left, Bottom	Left, Top
S05 PosN 105/100/110 4-5mm 4	2d (7.5 mg)	20250312-211540	172.461	1.027	0.22	0.22	0.1	y	46	0.005	26	0.004	4	3.5	-0.105	Left, Bottom	Left, Top
S05 PosN 105/100/110 4-5mm 5	2d (7.5 mg)	20250312-221802	173.501	1.033	0.22	0.22	0.14	y	46	0.005	36	0.004	4	3.5	-0.105	Left, Bottom	Left, Top
S05 PosN 105/100/110 4-5mm 6	2d (7.5 mg)	20250312-235242	175.078	1.042	0.22	0.22	0.1	y	46	0.005	26	0.004	4	3.5	-0.105	Left, Bottom	Left, Top
S05 PosN 105/100/110 4-5mm 7	2d (7.5 mg)	20250313-001539	175.461	1.044	0.22	0.22	0.1	y	46	0.005	26	0.004	4	3.5	-0.105	Left, Bottom	Left, Top
S05 PosN 105/100/110 4-5mm 8	2d (7.5 mg)	20250318-021622	297.473	1.771	0.22	0.22	0.26	y	46	0.005	53	0.005	4	3.5	-0.1	Left, Bottom	Left, Top
S05 PosN 105/100/110 4-5mm 9	2d (7.5 mg)	20250722-213851	3340.848	19.886	0.22	0.22	0.26	y	46	0.005	53	0.005	4	3.5	-0.1	Left, Bottom	Left, Top
S05 PosN 105/100/110 4-5mm 10	2d (7.5 mg)	20250722-215419	3341.105	19.888	0.22	0.22	0.1	y	46	0.005	26	0.004	4	3.5	-0.1	Left, Bottom	Left, Top
S05 PosN 105/100/110 4-5mm 11	2d (7.5 mg)	20250724-005132	3368.059	20.048	0.22	0.22	0.1	y	46	0.005	26	0.004	4	3.5	-0.1	Left, Bottom	Left, Top
S04 Pos1 110 1	2d (15 mg)	20250429-211219	5.172	0.031	0.22	0.22	0.18	y	46	0.005	37	0.005	4	2.8	-0.11	Left, Bottom	Left, Top
S04 Pos1 110 2	2d (15 mg)	20250430-205221	28.839	0.172	0.22	0.22	0.28	y	46	0.005	58	0.005	4	2.8	-0.11	Left, Bottom	Left, Top
S04 Pos1 110 3	2d (15 mg)	20250501-195511	51.886	0.309	0.22	0.22	0.28	y	46	0.005	58	0.005	4	2.8	-0.11	Left, Bottom	Left, Top
S04 Pos1 110 4	2d (15 mg)	20250723-223546	2046.563	12.182	0.2375	0.22	0.28	y	50	0.005	58	0.005	4	2.8	-0.11	Left, Bottom	Left, Top
S04 Pos3 110 1	2d (15 mg)	20250429-205620	4.906	0.029	0.22	0.22	0.18	y	46	0.005	37	0.005	4	2.8	-0.11	Left, Bottom	Left, Top
S04 Pos3 110 2	2d (15 mg)	20250430-203259	28.516	0.17	0.22	0.22	0.28	y	46	0.005	58	0.005	4	2.8	-0.11	Left, Bottom	Left, Top
S04 Pos3 110 3	2d (15 mg)	20250430-223403	30.534	0.182	0.22	0.22	0.28	y	46	0.005	58	0.005	4	2.8	-0.11	Left, Bottom	Left, Top
S04 Pos3 110 4	2d (15 mg)	20250501-174207	49.669	0.296	0.22	0.22	0.28	y	46	0.005	58	0.005	4	2.8	-0.11	Left, Bottom	Left, Top
S04 Pos3 110 5	2d (15 mg)	20250723-231853	2047.281	12.186	0.2375	0.22	0.28	y	50	0.005	58	0.005	4	2.8	-0.11	Left, Bottom	Left, Top
S04 PosN 110 1	2d (15 mg)	20250724-163807	2064.602	12.289	0.2375	0.22	0.28	y	50	0.005	58	0.005	4	2.8	-0.11	Left, Bottom	Left, Top
S04 PosN 110 2	2d (15 mg)	20250723-234122	2047.656	12.188	0.2375	0.22	0.28	y	50	0.005	58	0.005	4	2.8	-0.11	Left, Bottom	Left, Top
S04 PosN 110 3	2d (15 mg)	20250723-221616	2046.238	12.18	0.2375	0.22	0.28	y	50	0.005	58	0.005	4	2.8	-0.11	Left, Bottom	Left, Top
S04 PosN 110 4	2d (15 mg)	20250723-215208	2045.836	12.178	0.2375	0.22	0.28	y	50	0.005	58	0.005	4	2.4	-0.11	Left, Bottom	Left, Top
S04 PosN 110 5	2d (15 mg)	20250506-223107	174.485	1.039	0.2375	0.2375	0.28	y	50	0.005	58	0.005	4	2.8	-0.11	Left, Bottom	Left, Top
S04 PosN 110 6	2d (15 mg)	20250506-213352	173.531	1.033	0.2375	0.22	0.18	y	50	0.005	37	0.005	4	2.8	-0.11	Left, Bottom	Left, Top
S04 PosN 110 7	2d (15 mg)	20250501-213518	53.555	0.319	0.22	0.22	0.28	y	46	0.005	58	0.005	4	2.8	-0.11	Left, Bottom	Left, Top
S04 PosN 110 8	2d (15 mg)	20250501-171041	49.145	0.293	0.22	0.22	0.28	y	46	0.005	58	0.005	4	2.8	-0.11	Left, Bottom	Left, Top
S04 PosN 110 9	2d (15 mg)	20250430-233117	31.488	0.187	0.22	0.22	0.28	y	46	0.005	58	0.005	4	2.4	-0.11	Left, Bottom	Left, Top
S04 PosN 110 10	2d (15 mg)	20250430-213808	29.602	0.176	0.22	0.22	0.18	y	46	0.005	37	0.005	4	2.8	-0.11	Left, Bottom	Left, Top
S04 PosN 110 11	2d (15 mg)	20250430-165533	24.893	0.148	0.22	0.22	0.28	y	46	0.005	58	0.005	4	2.8	-0.11	Left, Bottom	Left, Top
S04 PosN 110 12	2d (15 mg)	20250430-163514	24.554	0.146	0.22	0.22	0.28	y	46	0.005	58	0.005	4	2.4	-0.11	Left, Bottom	Left, Top
S04 PosN 110 13	2d (15 mg)	20250429-215150	5.831	0.035	0.22	0.22	0.28	y	46	0.005	58	0.005	4	2.4	-0.11	Left, Bottom	Left, Top
S04 PosN 110 14	2d (15 mg)	20250429-195005	3.801	0.023	0.22	0.22	0.28	y	46	0.005	58	0.005	4	2.4	-0.11	Left, Bottom	Left, Top
100:1 ferucarbotran styrofoam head	4	20250913-002222	N/A	N/A	0.22	0.22	0.28	y	46	0.005	58	0.005	4	3.5	-0.09	Left, Bottom	Left, Top
100:1 ferumoxytol styrofoam head	4	20250905-015110	N/A	N/A	0.22	0.22	0.28	y	46	0.005	58	0.005	4	3.5	-0.09	Left, Bottom	Left, Top
ferucarbotran Derenzo XY	EDF 4b	20250925-224333	N/A	N/A	0.24	0.2375	0.05	y	50	0.005	21	0.003	4	3.5	0	N/A	N/A
ferucarbotran Derenzo XZ 1	EDF 4b	20250926-181933	N/A	N/A	0.24	0.2375	0.24	y	50	0.005	49	0.005	4	3.5	0	N/A	N/A
ferucarbotran Derenzo XZ 2	EDF 4b	20250926-180222	N/A	N/A	0.24	0.2375	0.24	y	50	0.005	49	0.005	4	3.5	0	N/A	N/A
ferumoxytol Derenzo XY	EDF 4c	20250524-010427	N/A	N/A	0.24	0.2375	0.04	y	50	0.005	17	0.003	4	3.5	0	N/A	N/A
ferumoxytol Derenzo XZ 1	EDF 4c	20250524-001401	N/A	N/A	0.24	0.2375	0.24	y	50	0.005	49	0.005	4	3.5	0	N/A	N/A
ferumoxytol Derenzo XZ 2	EDF 4c	20250523-235544	N/A	N/A	0.24	0.2375	0.24	y	50	0.005	49	0.005	4	3.5	0	N/A	N/A
shinethrough	EDF 5e	20240830-210248	N/A	N/A	0.22	0.22	0.05	x	46	0.005	11	0.005	4	3.5	0	N/A	N/A
sensitivity ferucarbotran 70ug	EDF 5fg	20240906-191415	N/A	N/A	0.24	0.226	0.24	y	50	0.005	49	0.005	4	3.5	0	N/A	N/A
sensitivity ferucarbotran 35ug	EDF 5fg	20240906-194001	N/A	N/A	0.24	0.226	0.24	y	50	0.005	49	0.005	4	3.5	0	N/A	N/A
sensitivity ferucarbotran 18ug	EDF 5fg	20240906-200742	N/A	N/A	0.24	0.226	0.24	y	50	0.005	49	0.005	4	3.5	0	N/A	N/A
sensitivity ferucarbotran 9ug	EDF 5fg	20240906-203715	N/A	N/A	0.24	0.226	0.24	y	50	0.005	49	0.005	4	3.5	0	N/A	N/A
sensitivity ferucarbotran 4ug	EDF 5fg	20240906-210541	N/A	N/A	0.24	0.226	0.24	y	50	0.005	49	0.005	4	3.5	0	N/A	N/A
sensitivity ferucarbotran 2ug	EDF 5fg	20240906-213414	N/A	N/A	0.24	0.226	0.24	y	50	0.005	49	0.005	4	3.5	0	N/A	N/A
sensitivity ferumoxytol 800ug	EDF 5fg	20241121-212212	N/A	N/A	0.22	0.22	0.24	y	46	0.005	49	0.005	4	3.5	0	N/A	N/A
sensitivity ferumoxytol 400ug	EDF 5fg	20241121-215231	N/A	N/A	0.22	0.22	0.24	y	46	0.005	49	0.005	4	3.5	0	N/A	N/A
sensitivity ferumoxytol 200ug	EDF 5fg	20241121-221159	N/A	N/A	0.22	0.22	0.24	y	46	0.005	49	0.005	4	3.5	0	N/A	N/A

Supplementary Table ST1: Clinical scanner image scanning parameters and internal identifier (UID) (continued).

sensitivity ferumoxytol 100ug	EDF 5fg	20241121-222925	N/A	N/A	0.22	0.22	0.24	y	46	0.005	49	0.005	4	3.5	0	N/A	N/A
sensitivity ferumoxytol 50ug	EDF 5fg	20241121-224631	N/A	N/A	0.22	0.22	0.24	y	46	0.005	49	0.005	4	3.5	0	N/A	N/A
sensitivity ferumoxytol 25ug	EDF 5fg	20241121-230252	N/A	N/A	0.22	0.22	0.24	y	46	0.005	49	0.005	4	3.5	0	N/A	N/A
surface coil sensitivity ferucabotran 18ug	EDF 6c	20250222-013236	N/A	N/A	0.22	0.22	0.06	y	46	0.005	13	0.005	4	3.5	-0.07	Bilateral, Bottom	Bilateral, Top
surface coil sensitivity ferucabotran 9ug	EDF 6c	20250222-005241	N/A	N/A	0.22	0.22	0.06	y	46	0.005	13	0.005	4	3.5	-0.07	Bilateral, Bottom	Bilateral, Top
surface coil sensitivity ferucabotran 4ug	EDF 6c	20250222-001032	N/A	N/A	0.22	0.22	0.06	y	46	0.005	13	0.005	4	3.5	-0.07	Bilateral, Bottom	Bilateral, Top
surface coil sensitivity ferucabotran 2ug	EDF 6c	20250222-002458	N/A	N/A	0.22	0.22	0.06	y	46	0.005	13	0.005	4	3.5	-0.07	Bilateral, Bottom	Bilateral, Top
surface coil sensitivity ferucabotran 1ug	EDF 6c	20250222-003610	N/A	N/A	0.22	0.22	0.06	y	46	0.005	13	0.005	4	3.5	-0.07	Bilateral, Bottom	Bilateral, Top
surface coil sensitivity ferucabotran 500n	EDF 6c	20250222-004446	N/A	N/A	0.22	0.22	0.06	y	46	0.005	13	0.005	4	3.5	-0.07	Bilateral, Bottom	Bilateral, Top
8000:1 styrofoam head	EDF 6d	20250718-224247	N/A	N/A	0.22	0.22	0.28	y	46	0.005	58	0.005	4	3.5	-0.09	Left, Bottom	Left, Top
rotation1	EDF 7a	20250430-205221	N/A	N/A	0.22	0.22	0.28	y	46	0.005	58	0.005	4	2.8	-0.11	Left, Bottom	Left, Top
rotation2	EDF 7a	20250430-203259	N/A	N/A	0.22	0.22	0.28	y	46	0.005	58	0.005	4	2.8	-0.11	Left, Bottom	Left, Top
rotation3	EDF 7a	20250430-165533	N/A	N/A	0.22	0.22	0.28	y	46	0.005	58	0.005	4	2.8	-0.11	Left, Bottom	Left, Top
positioning1	EDF 7b	20250430-215227	N/A	N/A	0.22	0.22	0.18	y	46	0.005	37	0.005	4	2.8	-0.08	Left, Bottom	Left, Top
positioning2	EDF 7b	20250501-003546	N/A	N/A	0.22	0.22	0.18	y	46	0.005	37	0.005	4	2.8	-0.09	Left, Bottom	Left, Top
positioning3	EDF 7b	20250501-002016	N/A	N/A	0.22	0.22	0.18	y	46	0.005	37	0.005	4	2.8	-0.1	Left, Bottom	Left, Top
positioning4	EDF 7b	20250430-213808	N/A	N/A	0.22	0.22	0.18	y	46	0.005	37	0.005	4	2.8	-0.11	Left, Bottom	Left, Top
Tumor 300ug	EDF 8a	20250311-002955	N/A	N/A	0.22	0.22	0.2	y	46	0.005	41	0.005	4	3.5	0	N/A	N/A
Tumor 600ug	EDF 8a	20250311-012342	N/A	N/A	0.22	0.22	0.2	y	46	0.005	41	0.005	4	3.5	0	N/A	N/A
Tumor 1000ug	EDF 8a	20250311-014124	N/A	N/A	0.22	0.22	0.2	y	46	0.005	41	0.005	4	3.5	0	N/A	N/A
ExtFig8_Perfusion1	EDF 8b	20240401-210951	N/A	N/A	0.22	0.22	0.11	x	46	0.005	56	0.002	8	3.5	0	N/A	N/A
ExtFig8_Perfusion2	EDF 8b	20240401-213732	N/A	N/A	0.22	0.22	0.16	x	46	0.005	81	0.002	8	3.5	0	N/A	N/A
Embo1	EDF 8c	20241221-005327	N/A	N/A	0.2	0.2	0	y	20	0.01	150	0	16	3.5	0	N/A	N/A
Embo2	EDF 8c	20241221-005733	N/A	N/A	0.2	0.2	0	y	20	0.01	150	0	16	3.5	0	N/A	N/A
Embo3	EDF 8c	20241221-010216	N/A	N/A	0.2	0.2	0	y	20	0.01	150	0	16	3.5	0	N/A	N/A

Supplementary Table ST2: "Bluesteel" colormap #255 values, formatted for import into 3D Slicer[61] (label | name | R | G | B | alpha).

0 0 0 0 1 255
1 1 0 2 5 255
2 2 0 4 10 255
3 3 0 6 14 255
4 4 1 8 17 255
5 5 1 9 20 255
6 6 1 11 23 255
7 7 2 13 25 255
8 8 2 14 28 255
9 9 2 16 30 255
10 10 2 17 32 255
11 11 2 18 34 255
12 12 2 19 37 255
13 13 2 20 39 255
14 14 2 21 41 255
15 15 2 22 43 255
16 16 1 23 45 255
17 17 1 24 47 255
18 18 1 25 49 255
19 19 1 26 50 255
20 20 0 27 52 255
21 21 0 28 54 255
22 22 0 28 56 255
23 23 0 29 57 255
24 24 0 30 59 255
25 25 0 31 61 255
26 26 0 32 62 255
27 27 0 33 64 255
28 28 0 34 66 255
29 29 0 34 67 255
30 30 0 35 69 255
31 31 0 36 71 255
32 32 0 37 72 255
33 33 0 38 74 255
34 34 0 39 76 255
35 35 0 40 77 255
36 36 0 40 79 255
37 37 0 41 81 255
38 38 0 42 82 255
39 39 0 43 84 255
40 40 0 44 86 255
41 41 0 45 87 255
42 42 0 45 89 255
43 43 0 46 90 255
44 44 0 47 92 255
45 45 1 48 93 255
46 46 1 49 94 255
47 47 1 50 96 255
48 48 2 51 97 255
49 49 2 52 98 255
50 50 3 53 99 255
51 51 3 53 100 255
52 52 4 54 101 255
53 53 4 55 102 255
54 54 5 56 103 255
55 55 6 57 104 255
56 56 6 58 105 255
57 57 7 59 106 255
58 58 8 60 106 255
59 59 9 61 107 255
60 60 9 62 108 255
61 61 10 63 109 255
62 62 11 64 109 255
63 63 12 65 110 255
64 64 12 66 111 255
65 65 13 67 112 255
66 66 14 68 113 255
67 67 15 68 113 255
68 68 15 69 114 255
69 69 16 70 115 255
70 70 17 71 116 255
71 71 17 72 116 255
72 72 18 73 117 255
73 73 19 74 118 255
74 74 19 75 119 255
75 75 20 76 119 255
76 76 21 77 120 255
77 77 22 78 121 255
78 78 22 78 122 255
79 79 23 79 122 255
80 80 24 80 123 255
81 81 24 81 124 255
82 82 25 82 125 255
83 83 26 83 125 255
84 84 26 84 126 255
85 85 27 85 127 255
86 86 28 86 127 255
87 87 28 86 128 255
88 88 29 87 129 255
89 89 30 88 130 255
90 90 30 89 130 255
91 91 31 90 131 255
92 92 32 91 132 255
93 93 32 92 132 255
94 94 33 92 133 255
95 95 34 93 134 255
96 96 34 94 135 255
97 97 35 95 135 255
98 98 36 96 136 255
99 99 36 97 137 255
100 100 37 97 137 255
101 101 37 98 138 255
102 102 38 99 139 255
103 103 39 100 139 255
104 104 39 101 140 255
105 105 40 101 141 255
106 106 41 102 141 255
107 107 41 103 142 255
108 108 42 104 143 255
109 109 43 105 143 255
110 110 43 106 144 255
111 111 44 106 145 255
112 112 44 107 145 255
113 113 45 108 146 255
114 114 46 109 147 255
115 115 46 110 147 255
116 116 47 110 148 255
117 117 47 111 149 255
118 118 48 112 149 255
119 119 49 113 150 255
120 120 49 113 151 255
121 121 50 114 151 255
122 122 50 115 152 255
123 123 51 116 152 255
124 124 52 116 153 255
125 125 52 117 154 255
126 126 53 118 154 255
127 127 54 119 155 255
128 128 54 120 155 255
129 129 55 120 156 255
130 130 56 121 156 255
131 131 57 122 157 255
132 132 58 123 157 255
133 133 59 123 157 255
134 134 60 124 157 255
135 135 61 125 157 255
136 136 63 126 156 255
137 137 65 126 156 255
138 138 67 127 155 255
139 139 69 128 154 255
140 140 71 129 152 255
141 141 73 129 151 255
142 142 76 130 150 255
143 143 78 131 148 255
144 144 80 132 146 255
145 145 83 132 145 255
146 146 85 133 143 255
147 147 88 134 141 255
148 148 90 134 140 255
149 149 92 135 138 255
150 150 95 136 136 255
151 151 97 136 134 255
152 152 99 137 133 255
153 153 102 138 131 255
154 154 104 138 129 255
155 155 106 139 128 255
156 156 108 140 126 255
157 157 111 140 124 255
158 158 113 141 123 255
159 159 115 142 121 255
160 160 117 142 120 255
161 161 119 143 118 255
162 162 122 144 116 255
163 163 124 144 115 255
164 164 126 145 113 255
165 165 128 145 112 255
166 166 130 146 110 255
167 167 132 147 108 255
168 168 134 147 107 255
169 169 137 148 105 255
170 170 139 148 104 255
171 171 141 149 102 255
172 172 143 150 101 255
173 173 145 150 99 255
174 174 147 151 98 255
175 175 149 152 96 255
176 176 151 152 95 255
177 177 153 153 93 255
178 178 156 153 92 255
179 179 158 154 90 255

1303 180 180 160 155 89 255
1304 181 181 162 155 87 255
1305 182 182 164 156 86 255
1306 183 183 166 156 84 255
1307 184 184 168 157 83 255
1308 185 185 170 158 81 255
1309 186 186 172 158 80 255
1310 187 187 174 159 78 255
1311 188 188 177 159 77 255
1312 189 189 179 160 75 255
1313 190 190 181 160 74 255
1314 191 191 183 161 72 255
1315 192 192 185 162 71 255
1316 193 193 187 162 69 255
1317 194 194 189 163 68 255
1318 195 195 191 163 66 255
1319 196 196 193 164 64 255
1320 197 197 195 165 63 255
1321 198 198 198 165 61 255
1322 199 199 200 166 60 255
1323 200 200 202 166 58 255
1324 201 201 204 167 57 255
1325 202 202 206 168 55 255
1326 203 203 208 168 54 255
1327 204 204 210 169 52 255
1328 205 205 212 170 51 255
1329 206 206 215 170 49 255
1330 207 207 217 171 48 255
1331 208 208 219 171 46 255
1332 209 209 221 172 45 255
1333 210 210 223 173 43 255
1334 211 211 225 173 42 255
1335 212 212 227 174 40 255
1336 213 213 230 174 39 255
1337 214 214 232 175 38 255
1338 215 215 234 176 36 255
1339 216 216 236 176 35 255
1340 217 217 238 177 34 255
1341 218 218 240 178 34 255
1342 219 219 241 179 34 255
1343 220 220 243 180 35 255
1344 221 221 244 181 36 255
1345 222 222 245 182 38 255
1346 223 223 246 183 41 255
1347 224 224 247 184 45 255
1348 225 225 248 185 49 255
1349 226 226 248 187 54 255
1350 227 227 248 188 59 255
1351 228 228 248 190 65 255
1352 229 229 248 191 71 255
1353 230 230 247 193 77 255
1354 231 231 247 195 83 255
1355 232 232 246 196 90 255
1356 233 233 245 198 96 255
1357 234 234 245 200 103 255
1358 235 235 244 201 110 255
1359 236 236 243 203 116 255
1360 237 237 243 204 123 255
1361 238 238 242 206 130 255
1362 239 239 241 208 136 255
1363 240 240 240 209 143 255
1364 241 241 240 211 149 255
1365 242 242 239 212 156 255
1366 243 243 238 214 162 255
1367 244 244 237 215 168 255
1368 245 245 237 217 174 255
1369 246 246 236 218 181 255
1370 247 247 235 220 187 255
1371 248 248 235 221 193 255
1372 249 249 234 222 199 255
1373 250 250 233 224 205 255
1374 251 251 232 225 211 255
1375 252 252 232 227 217 255
1376 253 253 231 228 223 255
1377 254 254 230 229 229 255

Supplementary Videos SV1-SV2

Supplementary Video SV1: Video of tomographic MPI images co-registered with de-indentified surface scans (see **Figure 1f,g**).
File: *SV1.mp4*.

Supplementary Video SV2: Real-time 2D axial slice imaging at 0.8 frames/second of a trans-arterial embolization liver phantom being filled with ferucarbotran (see **Extended Data Figure 8b**).
File: *SV2.mp4*.

Supplementary Data SD1-SD2

Supplementary Data SD1: 3D Slicer[61] scene for tomographic MPI image co-registered with de-indentified surface scan. Image of subject with 7.5 mg ferumoxytol injection at t = 12.4 days, with two fiducials (Fid) containing 0.5 mg ferumoxytol (see **Figure 1f**).
File: *SD1_scene_S05_2025-08-12.mrb*.

Supplementary Data SD2: 3D Slicer[61] scene for tomographic MPI image co-registered with de-indentified surface scan. Image of subject with 15 mg ferumoxytol injection at t = 28.5 hours, no fiducials used (see **Figure 1g**).
File: *SD2_Scene_S04_2025-08-19.mrb*.

Supplementary Files

This is a list of supplementary files associated with this preprint. Click to download.

- SV1.mp4
- SV2.mp4
